# Two-Dimensional Tantalum Carbo-Selenide for Hydrogen Evolution

**DOI:** 10.1021/acsnano.4c09903

**Published:** 2025-01-16

**Authors:** Elham Loni, Ahmad Majed, Shengjie Zhang, Hari H. S. Thangavelu, Chaochao Dun, Anika Tabassum, Karamullah Eisawi, Jeffrey J. Urban, Per O. Å. Persson, Matthew M. Montemore, Michael Naguib

**Affiliations:** † Department of Physics and Engineering Physics, Tulane University, New Orleans, Louisiana 70118, United States; ‡ Department of Chemical and Biomolecular Engineering, Tulane University, New Orleans, Louisiana 70118, United States; § Department of Physics, Chemistry and Biology, Linköping University, Linköping SE-581 83, Sweden; ∥ The Molecular Foundry, Lawrence Berkeley National Laboratory, Berkeley, California 94720, United States

**Keywords:** two-dimensional materials, transition metal carbo-chalcogenides, delamination, hydrogen evolution, electrocatalyst

## Abstract

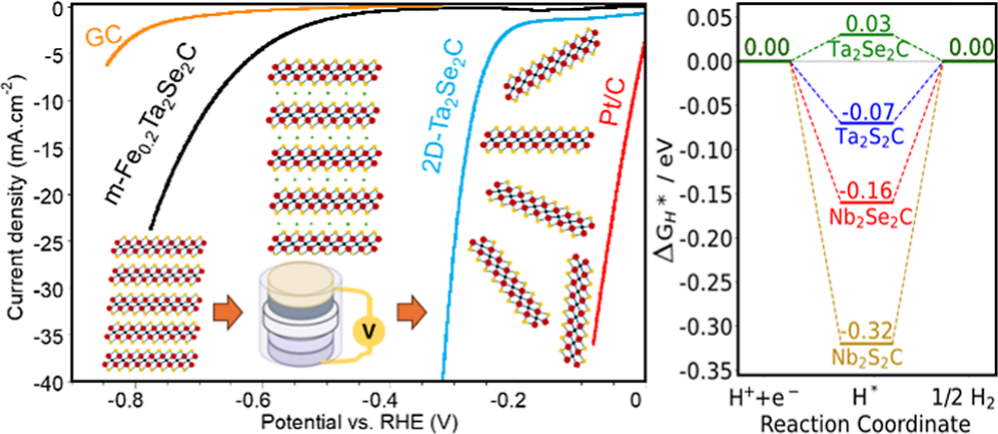

Herein, we report the synthesis of two-dimensional Ta_2_Se_2_C (2D-Ta_2_Se_2_C) nanosheets using electrochemical lithiation in multilayer Ta_2_Se_2_C followed by sonication in deionized water. Multilayer Ta_2_Se_2_C was obtained via solid-state synthesis of Fe_*x*_Ta_2_Se_2_C followed by chemical etching of Fe. 2D-Ta_2_Se_2_C exhibited promising electrocatalytic activity for the hydrogen evolution reaction from water compared to multilayer Ta_2_Se_2_C and 2D-TaSe_2_. 2D-Ta_2_Se_2_C showed an overpotential at 10 mA·cm^–2^ (η_10_) of 264 mV, a Tafel slope of 91 mV·dec^–1^, and an electrochemically active surface area of 17.61 m_ECSA_^2^·g_catalyst_^–1^. The high performance could be attributed to the large surface area of single sheets which hence maximizes the number of exposed catalytic sites and increased density of vacancies, observed with transmission electron microscopy, during synthesis and processing.

Transition metal dichalcogenides (TMDs) are layered structures consisting of a core transition metal layer bonded to two surface layers of chalcogen atoms with a general chemical formula of MX_2_ (M: transition metal, X: chalcogen such as S, Se, or Te).^1^ These materials have direct bandgaps and interesting electronic and optical properties, which make them suitable candidates for various applications such as electronics^2^ and optoelectronics.^3^ Monolayers of TMDs were shown to be catalytically active with different electrical properties ranging from insulating to semiconducting and metallic, leading to their applications in electrochemical energy storage,^4^ electrocatalysis,^5,6^ heterogeneous catalysis,^7^ and photocatalysis.^8^ Especially 1T and 1T′ phases showed high electrical conductivity and have been reported as outstanding electrocatalysts for the hydrogen evolution reactions (HERs). For example, Lukowski et al.^9^ reported the synthesis of 1T-MoS_2_ using lithium intercalation and chemical exfoliation of 2H–MoS_2_ that showed a low overpotential of 187 mV for a current density of 10 mA·cm^–2^. In another study, Voiry et al. reported exfoliation of 1T-MoS_2_ nanosheets with a Tafel slope of 40 mV·dec^–1^.^10^ TMDs are known to be earth-abundant materials as potential replacements for noble metal-based catalysts in the electrocatalytic HER,^5,11,12^ due to their unique atomic structure and optimized active site density and surface area. However, these 2D-TMDs suffer from poor stability when exposed to ambient conditions. These materials, particularly in their monolayer or few-layer forms, are prone to oxidation and degradation upon exposure to air and moisture, which can significantly alter their electronic properties and hinder their performance in different applications.^13^ For example, oxidation can result in the formation of metal oxides, disrupting the pristine crystalline structure and impairing conductivity. Additionally, defect sites in the crystal lattice, such as vacancies or grain boundaries, can exacerbate the degradation process, making these materials more susceptible to environmental stressors. Various approaches, such as encapsulation with stable 2D materials (e.g., graphene or hexagonal boron nitride) or functionalization with protective coatings, are being explored to mitigate these stability issues. Improving the stability of 2D-TMDs remains a critical challenge for their use in long-term and practical applications like flexible electronics and optoelectronic devices.^13,14^ An alternative approach is the synthesis of new 2D materials that have high electrical conductivity, high catalytic activity, and higher stability at the same time. For instance, if we can synthesize 2D layered materials with a chalcogenide surface like TMDs and the carbide core of MXenes,^15^ the resulting material could have extraordinary electrocatalytic performance owing to the combination of high catalytic activity, electrical conductivity, and high stability.

Recently, we reported on the large-scale synthesis of layered transition metal carbo-chalcogenides (TMCCs) and their exfoliation, including 2D-Ta_2_S_2_C and Nb_2_S_2_C, with superconductivity characteristics, high elastic constants, and promising performance as electrode materials in Li-ion batteries. TMCCs (M_2_X_2_C; M: transition metal, X: chalcogen, C: carbon) are a family that exhibits different properties and applications by tuning their composition.^16^ This family has the potential to expand by using different transition metals, as well as different chalcogens. The first successful synthesis of the TMCC multilayer was reported for Ta_2_S_2_C in 1970 by Beckmann et al.^17^ Later, more reports on the synthesis of TMCCs were published, such as M_*x*_Nb_2_S_2_C (M: V, Cr, Mn, Fe, Co, Ni, Cu)^18^ and Nb_2_S_2_C.^19^ Recently, many *ab initio* calculation studies have focused on predicting the properties of various TMCCs.^20−25^ These materials have layered structures with strong covalent intralayer bonding and relatively weak van der Waals out-of-plane bonding,^25^ which makes their exfoliation process possible, as reported for the first time for 2D-Ta_2_S_2_C and Nb_2_S_2_C by Majed et al. in 2022.^16^

Although theoretical calculations have predicted the stability and properties of many 2D-TMCCs, most experimental studies have only focused on structures with sulfur as the chalcogen. There are still many TMCCs that have never been synthesized or reported, such as those including selenium, tellurium, and/or a solid solution of sulfur, selenium, and/or tellurium.

Herein, we report the synthesis of 2D Ta_2_Se_2_C. As shown in Scheme 1, multilayered Ta_2_Se_2_C was produced by the solid-state synthesis of Fe_*x*_Ta_2_Se_2_C, followed by chemical etching of iron. To exfoliate these materials, lithium (Li) was electrochemically intercalated between the layers (electrochemical cell schematic is shown in Scheme S1). The Li-intercalated Ta_2_Se_2_C was then submerged in water and sonicated to induce swelling and exfoliation of the layers. 2D-Ta_2_Se_2_C exhibited superior performance as an electrocatalyst for HER compared to their multilayer counterparts. Density functional theory (DFT) calculations were performed to predict the possible active sites of Ta_2_Se_2_C and three other TMCCs for comparison.

**Scheme 1:**
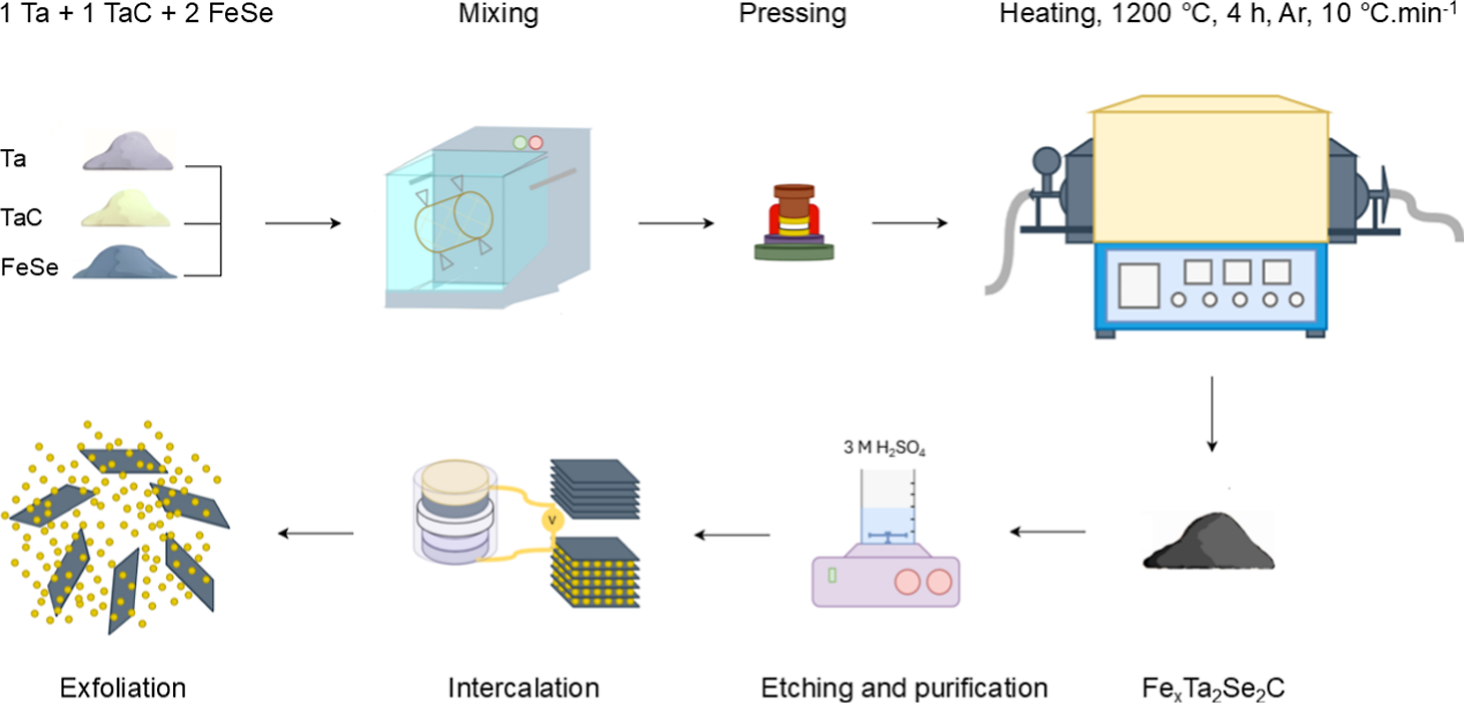
Schematic Illustration of the Synthesis Procedure of 2D-Ta_2_Se_2_C

## Results and Discussion

### Characterization of Materials

**Figure 1:**
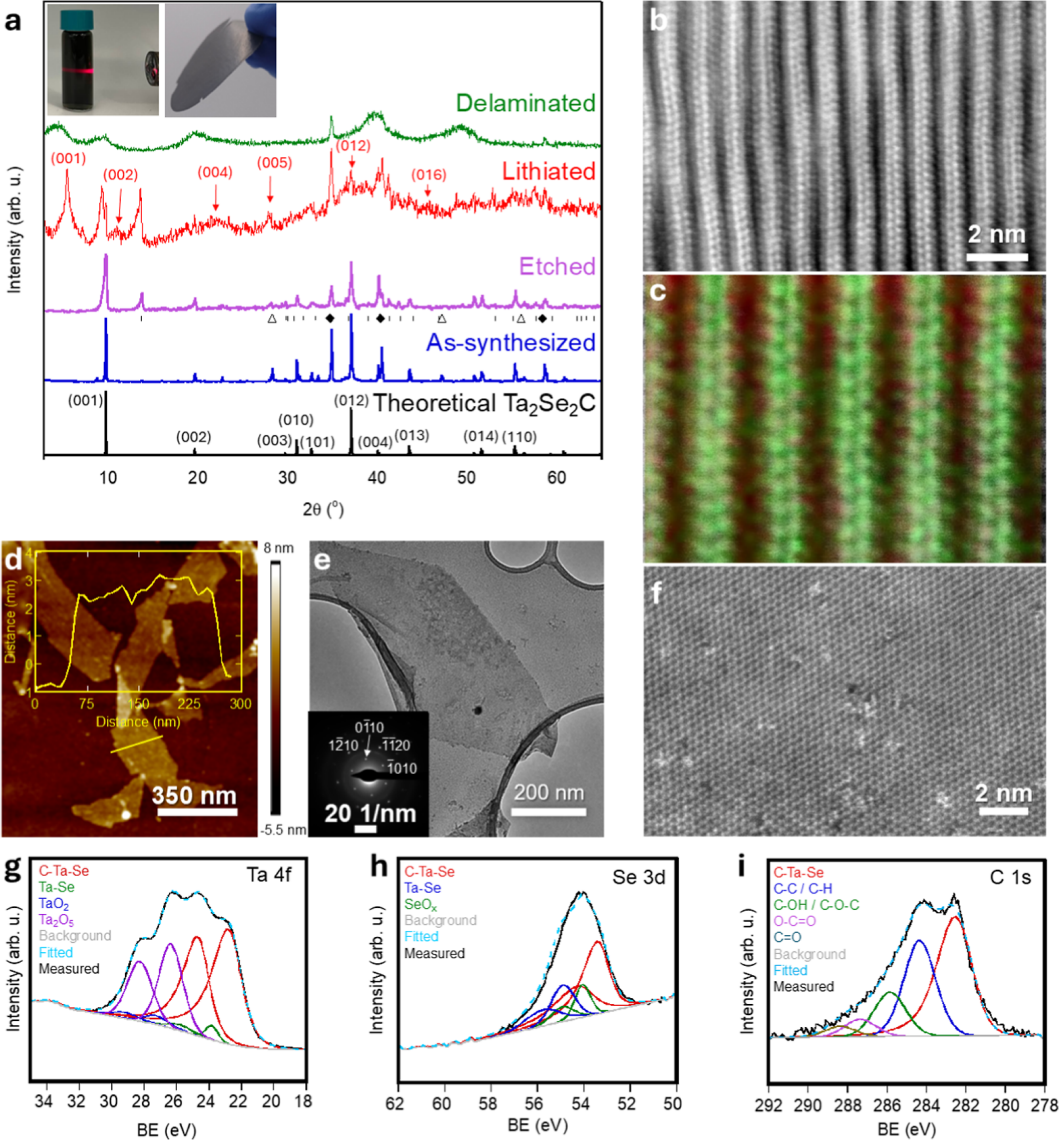
(a) XRD patterns of theoretical Ta_2_Se_2_C (black) and experimentally measured as-synthesized multilayer Fe_*x*_Ta_2_Se_2_C before (blue) and after (purple) etching, then lithiated (red), and delaminated (green) Ta_2_Se_2_C. The peaks identified by ⧫ indicate peaks for the TaC phase (PDF#00–035–0801), | indicate peaks for the TaSe_2_ phase (PDF#21–1200), and Δ referred to Si used as reference. Insets: left, the Tyndall effect of the colloidal dispersion after delamination, and right, free-standing paper. (b) HAADF–STEM image of Ta_2_Se_2_C and (c) its corresponding EELS map overlay (green: Ta, red: Se), (d) AFM image of the 2D-Ta_2_Se_2_C few-layer sheets. Inset: height profile along the identified yellow line. (e) TEM image of 2D-Ta_2_Se_2_C single sheets. Inset: corresponding SAED pattern. (f) Atomically resolved STEM plan-view image of the 2D-Ta_2_Se_2_C. (g–i) XPS spectra of the Ta 4f, C 1s, and Se 3d regions, respectively, for 2D-Ta_2_Se_2_C.

Figure 1a shows the X-ray diffraction (XRD) patterns of the as-synthesized, etched, lithiated, and delaminated samples. FeSe was used as a source for Se to enable synthesis at temperatures above the boiling point of Se, similar to our previous work on Ta_2_S_2_C,^16^ where FeS was used as a source for S and similar to what Chen et al.^26^ reported for Ti_2_SC. This approach allowed us to use a tube furnace at ambient pressure, avoiding the need to seal the precursors in quartz tubes under vacuum and limiting the heating to temperatures below 1200 °C. Solid-state synthesis of Fe_*x*_Ta_2_Se_2_C with the highest possible purity was crucial for the successful synthesis of the corresponding multilayer and delaminated Ta_2_Se_2_C. Details on the different synthesis parameters (precursors composition, time, and temperature) considered in the synthesis of Fe_*x*_Ta_2_Se_2_C are provided in Supporting Information (Table S1 and Figure S1). We found that the optimum conditions for achieving the highest content of Fe_*x*_Ta_2_Se_2_C, relative to the secondary phases of Fe, TaC, and Fe_*x*_TaSe_2_, are a molar ratio of Ta/TaC/FeSe = 1.0:1.0:2.0 and a heating to 1200 °C for 4 h under continuous Ar flow. A characteristic XRD peak for Fe_*x*_Ta_2_Se_2_C with the *P*3̅*m*1 space group was found at a 2θ of ∼9.96°, which is very close to what was reported for Fe_*x*_Ta_2_S_2_C.^16^

To remove the secondary phase of iron as well as intercalated iron from between the layers, we used a H_2_SO_4_ treatment. To confirm the removal of Fe, we used an EDS analysis (Table S3). The results showed a drop in the Fe: Ta atomic ratio from 1.00:1.00 to 0.09:1.00 after H_2_SO_4_ treatment, proving Fe was removed mostly from the sample. Hence, Fe_0.2_Ta_2_Se_2_C is used as the chemical formula for the multilayer sample after etching. Scanning electron microscopy (SEM) images of the particles before (Figure S4a,b) and after (Figure S4c,d) etching showed that both samples exhibit layered morphologies.

Predicting that both Ta_2_Se_2_C and Ta_2_S_2_C have similar crystal structures but slightly different lattice parameters, they are expected to exhibit similar XRD patterns with slight shifts in the diffraction peaks’ positions. Thus, we used the structure of the Ta_2_S_2_C phase (PDF#00–024–1258) and replaced S with Se to predict the XRD pattern of Ta_2_Se_2_C. Using Bragg’s law, the *d*_spacing_ of the different crystallographic planes in Ta_2_Se_2_C was calculated, and from there, the lattice parameters were calculated. The *a* and *c* lattice parameters were calculated to be 3.29 and 8.93 ± 0.05 Å, respectively. The XRD pattern based on these calculated lattice parameters was simulated using VESTA. The details for the peaks’ positions and their corresponding crystallographic planes for the m-Fe_0.2_Ta_2_Se_2_C sample and the calculated data are provided in Table S2. To obtain a precise quantification for the phases present in the sample synthesized by the solid-state procedure as well as the lattice parameters, Rietveld refinement was employed (Supporting Information). According to the Rietveld refinement results, the *c*-lattice parameter stayed almost unchanged after the H_2_SO_4_ treatment (Δ*c* ≈ 0.02 Å decrease). We anticipated a decrease in the interlayer spacing after etching due to the removal of the Fe atoms from between the Ta_2_Se_2_C layers without the formation of new surface terminations as Ta atoms are already terminated by Se during the solid-state synthesis. This contrasts with typical 2D MXenes’ synthesis, where the etching process (e.g., removal of Al from the Ti_2_AlC MAX phase to form MXene) is accompanied by the introduction of new surface terminations, such as –OH, –O, or –F groups, which leads to an increase in interlayer spacing compared to the parent MAX phases. The behavior of Fe-intercalated Ta_2_Se_2_C is predicted to be more analogous to that of intercalated MXenes rather than MAX phases. For intercalated MXenes, deintercalation results in a decrease in interlayer spacing.^27,28^ The small change in the *c*-lattice parameter after H_2_SO_4_ treatment suggests that the presence of a small amount of iron might be sufficient to maintain the interlayer spacing for the Ta_2_Se_2_C structure; similar behavior was reported for intercalated 2D materials where a small amount of intercalants can act as pillars maintaining the interlayer spacing.^27−30^

XRD after etching revealed a more pronounced peak at 2θ of 13.86° that can be assigned to TaSe_2_ (PDF#21–1200). This phase already existed in the Fe_*x*_Ta_2_Se_2_C sample, but after etching and purification, its peaks grew in intensity.

A high-resolution cross-sectional high-angle annular dark field (HAADF)–scanning transmission electron microscopy (STEM) image of a m-Fe_0.2_Ta_2_Se_2_C particle together with a corresponding electron energy loss spectroscopy (EELS) elemental map overlay are shown in Figure 1b,c. These images illustrate layered sheets that consist of two atomic layers of tantalum sandwiched between two atomic layers of selenium. The EELS quantification revealed a Ta: Se molar ratio of 2.0:1.8 (Table S4 and Figure S5a), where the deviation from the 2.0:2.0 ratio can be explained by the formation of Se vacancies during acid etching. The interlayer spacing determined from the HAADF–STEM image was found to be 0.98 ± 0.02 nm. The slight difference between TEM (0.98 nm) and XRD (0.89 nm) interlayer spacings likely arises from the localized nature of TEM compared to the bulk averaging of XRD, as well as peak broadening or asymmetry in the XRD data due to sample inhomogeneity or restacking.

The XRD pattern of Li/*m*-Fe_0.2_Ta_2_Se_2_C after electrochemical lithiation (voltage profile is shown in Figure S3) indicated a significant shift in the (00*l*) peaks toward lower angles (Figure 1a). The (001) peak at a 2θ of 9.96° shifted to about 5.52°, indicating an increase in *d*_spacing_ of 0.71 nm, which is due to the intercalation of Li between the layers. Here, we also noticed the peaks related to m-Fe_0.2_Ta_2_Se_2_C (at 2θ ∼ 9.8°) and TaSe_2_ (at 2θ ∼ 13.8°) structures, suggesting the presence of some unintercalated m-Fe_0.2_Ta_2_Se_2_C and TaSe_2_ in the sample. After Li-intercalation, deionized (DI) water was added to the sample while starting sonication. The addition of water results in the formation of LiOH (which is water-soluble) and H_2_ gas. The mechanical force from ultrasonic-induced cavitation of the H_2_ bubbles leads to the exfoliation of the layers.^28^ After exfoliation, centrifuging the sample led to the separation of the few-layer sheets in the supernatant from multilayer particles and secondary phases in the precipitate. The inset in Figure 1a shows the supernatant exhibiting the Tyndall effect, suggesting the formation of a colloidal dispersion and a free-standing paper obtained after vacuum-assisted filtration of the supernatant. A cross-sectional SEM image of the free-standing paper, shown in Figure S4g, reveals restacked layers due to vacuum-assisted filtration. XRD for the free-standing paper of 2D-Ta_2_Se_2_C (Figure 1a) shows a broadening and significant shifting of the early peak at 9.96° for m-Fe_0.2_Ta_2_Se_2_C toward a lower angle of about 4.68°, which is due to the exfoliation of the multilayer structure to monolayers. Observation of broad and low-intensity. XRD peaks at lower angles in the 2D-Ta_2_Se_2_C paper indicate a less ordered restacking of the delaminated layers during the vacuum-assisted filtration process used to form the free-standing paper. All of the peaks, as marked in the XRD pattern, correspond to different (00*l*) planes of the Ta_2_Se_2_C phase. The peak at 35.07° corresponds to (111) planes of the TaC phase (PDF#00–035–0801). Freeze-drying the supernatant results in the formation of an aerogel. The SEM images for the aerogel sample in Figure S4e,f indicate a flaky morphology in the microscale, suggesting single- and/or few-layer sheets indicating the success of the delamination step. Figure 1d illustrates a few-layer sheet of Ta_2_Se_2_C captured by AFM. The height profile for an identified scan line is shown in the inset of Figure 1d. From the height profile, the sheet thickness was determined to be <4 nm, which indicates the measured sheet has few layers of thickness.

To gain better insight into the morphology and structure of 2D-Ta_2_Se_2_C, we used TEM and selected-area electron diffraction (SAED). Figure 1e shows a TEM image of a single sheet and the corresponding SAED pattern indicating the hexagonal symmetry which means the material has preserved its structure through the lithiation and exfoliation processes.^16,31,32^ The SAED pattern of 2D-Ta_2_Se_2_C reveals a well-ordered hexagonal symmetry inherited from m-Fe_0.2_Ta_2_Se_2_C, characteristic of high crystallinity. The HAADF–STEM plan-view image of a single layer, shown in Figure 1f, reveals a honeycomb arrangement of atoms. This is directly related to the arrangement of the Ta atoms, which dominate the image contrast through the *Z*^2^ mechanism, in agreement with the SAED pattern. Figure S5b shows the individual and mixed elemental maps from STEM–EELS, with Ta in green and Se in red, revealing distinct alternating layers of Ta and Se, highlighting the layered structure characteristic of 2D-Ta_2_Se_2_C. Figure S5c,d presents a magnified view of the HAADF–STEM plan-view image of a single sheet of 2D-Ta_2_Se_2_C, along with its corresponding fast Fourier transform pattern. The atomic arrangement reveals a Ta–Ta spacing of 2.5 Å. Atomic defects such as vacancies and pinholes can be observed as dark spots, which can be useful as anchoring sites for single-atom catalysts as well as for tuning the material’s properties, similar to what has been reported for other 2D materials.^32−36^ More TEM images of 2D-Ta_2_Se_2_C are provided in Figure S6a–d.

We used XPS measurements to gain a better understanding of the chemical nature of the elements on the surface of the sample and the chemical composition of the 2D sample. The survey spectrum (Figure S7) from −10 to 1350 eV indicated the presence of Ta, Se, C, Fe, Li, and Cu in the sample. High-resolution XPS spectra for the Ta-4f region (Figure 1g) can be deconvoluted using 8 peaks. The doublets at 22.6 and 24.69 eV correspond to 4f_7/2_ and 4f_5/2_, respectively. These peaks were assigned to Ta_2_Se_2_C since they are located between the peaks for TaC^36^ and TaSe_2_.^37,38^ The peaks at 23.2 and 25.74 eV correspond to TaSe_2_.^37,38^ The peaks at 29.14 and 26.36 eV originated from TaO_2_^39^ and Ta_2_O_5_^40^ oxides with doublets at 28.27 and 29.14 eV, respectively. These oxides could have formed after the solid-state synthesis as native oxides like what has been reported for layered carbides,^41,42^ during etching, delamination, and/or sample preparation for XPS. The high-resolution XPS spectra for the Se-3d region (Figure 1h) can be fitted using 6 main peaks. The peaks at 53.4 and 54.3 eV correspond to 3d_5/2_ and 3d_3/2_, respectively. These peaks were assigned to Ta_2_Se_2_C.^16^ The other doublets at 54.05 and 54.91 eV are related to TaSe_2_. The peaks at 55.76 and 55.89 eV correspond to SeO_*x*_.^43,44^ TaSe_2_ was formed during the solid-state synthesis process and the SeO_*x*_ peak is related to surface oxidation that can occur during etching and or delamination, handling, and or sample preparation for XPS measurements, which leads to Se vacancy formation.^43^ Fitting the C 1s spectrum in Figure 1i resulted in five peaks. The peak at 282.6 eV was assigned to Ta_2_Se_2_C. The second peak at higher binding energy, located at 284.4 eV, is related to the C 1s peak for C–C and or C–H chemical bonds.^45^ The peaks at 285.9, 287.4, and 288.4 eV were related to C–OH or C–O–C, C=O, and O–C=O, respectively.^45,46^

The high-resolution XPS spectra of the Fe 2p region (Figure S8) were very weak, indicating the small amount of Fe in the sample with a Fe/Ta molar ratio of 0.196:1.00 obtained from XPS quantification (Table S5), and in agreement with EDS (Table S3) and inductively coupled plasma mass spectrometry (ICPMS) (Table S6) results. The observed increase in relative Fe content in the delaminated sample is likely due to the removal of TaC during the delamination process, which does not contain Fe. This results in a relative enrichment of Fe in the remaining sample despite the absolute Fe content remaining unchanged.

The Fe 2p region can be resolved into two main peaks corresponding to Fe^0^ and Fe^3+^ (Fe_2_O_3_) at 706.9 and 709.5 eV, respectively, along with their associated doublets at 719.7 and 722.7 eV, respectively. Since Li 1s overlaps with Se 3d, it was not possible to study Li using XPS. Thus, we used ICPMS (Table S6) to quantify the residual Li content in the 2D-Ta_2_Se_2_C sample and found the molar ratio of Li/Ta to be 0.38:1.00.

The UV–vis spectra for m-Fe_0.2_Ta_2_Se_2_C and 2D-Ta_2_Se_2_C in DI water are shown in Figure S10, clearly illustrating differences in absorption behavior. While both materials exhibit strong UV absorbance, 2D-Ta_2_Se_2_C retains significantly higher absorbance in the visible and near-infrared regions than m-Fe_0.2_Ta_2_Se_2_C and Ta_2_O_5_.^46^ According to the literature, Ta_2_O_5_^46,47^ displays minimal absorbance beyond the UV range, typical of a bulk wide-bandgap insulator. The enhanced visible and near-infrared absorbance in 2D-Ta_2_Se_2_C suggests quantum confinement effects or surface plasmon resonances, which are absent in the bulk-like m-Fe_0.2_Ta_2_Se_2_C and Ta_2_O_5_.^46,47^ The higher absorbance for 2D-Ta_2_Se_2_C across the visible spectrum, compared to both m-Fe_0.2_Ta_2_Se_2_C and Ta_2_O_5_,^46,47^ may be attributed to the unique electronic and structural properties introduced by the 2D exfoliation process, leading to increased light–matter interactions and potentially more active sites for electronic transitions.^48^ However, the semifeatureless UV–vis might be explained by the presence of other bulk phases coexisting with delaminated materials.^49^ These bulk phases are most likely TaC, which was detected in the XRD patterns after delamination and centrifugation. Additionally, a small amount of undelaminated m-Fe_0.2_Ta_2_Se_2_C particles which cannot be detected in the XRD patterns might also coexist with the delaminated materials. Thus, further systematic studies are required to purify the samples after delamination and to eliminate any bulk phases. Such studies might involve varying sonication time and frequency, centrifugation speed, and solvent type.^50^

The electrical conductivity values obtained using the four-probe method are listed in Table S7. For m-Fe_0.2_Ta_2_Se_2_C, the conductivity was found to be 2.75 S·cm^–1^, but it dropped to 0.46 S·cm^–1^ after delamination (2D-Ta_2_Se_2_C). However, 2D-Ta_2_Se_2_C shows a slightly higher electrical conductivity than 2D-TaSe_2_ (0.41 S·cm^–1^). Potentiostatic electrochemical impedance spectroscopy (PEIS) measurements (Figure S9e) reveal the same trend with lower resistance for m-Fe_0.2_Ta_2_Se_2_C (0.38 Ω·cm^2^) compared to that for 2D-Ta_2_Se_2_C (0.58 Ω·cm^2^) and 2D-TaSe_2_ (0.71 Ω·cm^2^). Here, we also see a slightly lower resistance for 2D-Ta_2_Se_2_C compared to that for 2D-TaSe_2_. This behavior is explained by the oxidation of samples during the etching and delamination, as well as the loss of van der Waals interactions between layers, reducing electron mobility, while selenium vacancies introduce midgap defect states that trap charge carriers and further hinder conductivity. These vacancies are particularly detrimental in both Ta_2_Se_2_C and TaSe_2_, correlating with the sharp decline in conductivity, as they disrupt the metallic interlayer coupling and charge transport pathways, a phenomenon observed across many TMD systems.^51^ However, multilayer and delaminated TaSe_2_ and Ta_2_Se_2_C samples show low electrical conductivities across all samples, largely probably due to selenium vacancies and oxidation of the samples introduced during the etching process used to remove intercalated Fe atoms.

### Electrocatalytic Performance for HER

**Figure 2:**
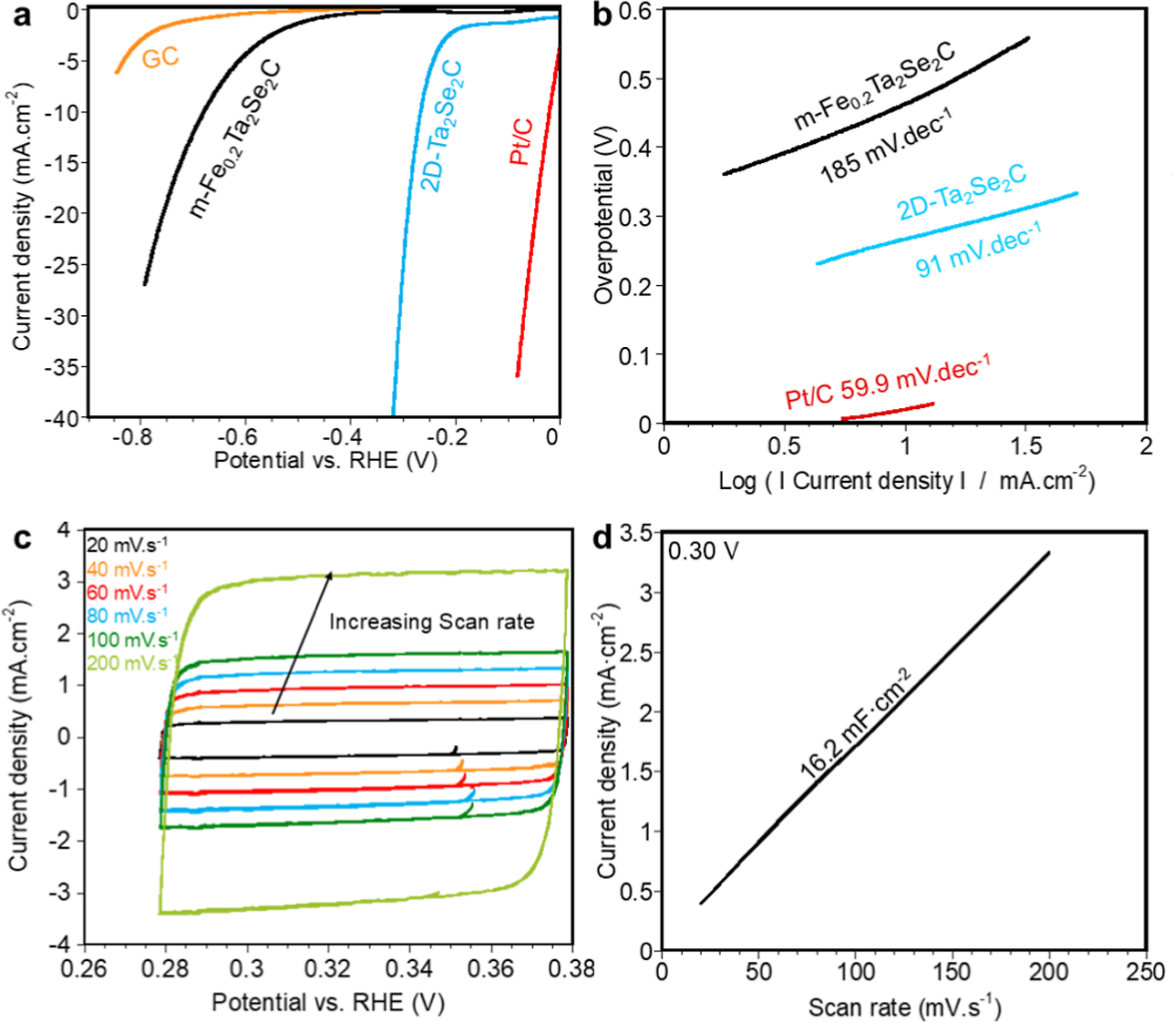
Electrochemical performance of Ta_2_Se_2_C as an electrocatalyst for HER. (a) LSV curves for glassy carboy (GC), Pt/C, m-Fe_0.2_Ta_2_Se_2_C, and 2D-Ta_2_Se_2_C with H_2_ bubbling after 20 CVs at OCP ± 50 mV in a 3-electrode cell with a 0.5 M H_2_SO_4_ electrolyte with Hg/Hg_2_SO_4_ in saturated K_2_SO_4_ as the reference electrode and Pt wire as the counter electrode. (b) Tafel polarization curves derived from LSV curves for m-Fe_0.2_Ta_2_Se_2_C and 2D-Ta_2_Se_2_C. (c) CVs at different scan rates of 20, 40, 60, 80, 100, and 200 mV·s^–1^ at the potential window of 0.28–0.38 V (OCP ± 50). (d) Current density versus scan rate plot corresponding to 0.30 V extracted from CVs at different scan rates.

Here, we focused on the electrochemical performance of Ta_2_Se_2_C as an electrocatalyst for HER. Figure 2a illustrates the linear sweep voltammetry (LSV) diagrams for m-Fe_0.2_Ta_2_Se_2_C and 2D-Ta_2_Se_2_C when used as an electrocatalyst for HER while bubbling H_2_. LSV values for glassy carbon (GC) and Pt/C electrodes were also plotted for comparison. The LSV diagrams were collected after 20 cyclic voltammograms (CVs) in the range of open-circuit potential (OCP) ± 50 mV to eliminate the effect of any possible reactions in the system during LSV measurements. The LSV was carried out in a 3-electrode cell using H_2_ gas bubbling. The overpotential (η_10_) of Ta_2_Se_2_C decreased from 678 to 264 mV due to delamination from m-Fe_0.2_Ta_2_Se_2_C to 2D-Ta_2_Se_2_C, respectively. The two distinct slopes in the LSV for the delaminated sample, with significant current observed in the 0 to −0.3 V range, indicate an increased surface area and a higher density of active sites compared to the multilayer sample. The early onset of current can be attributed to better accessibility to catalytic sites or surface defects introduced by the delamination process, as suggested by previous studies on the effect of increased surface area and delamination on catalytic performance.^52^ Since the Pt counter electrode might affect the accuracy of overpotential measurements, especially in acidic electrolytes,^53,54^ we repeated LSV measurements using a graphite rod counter electrode (Figure S9a). A slightly lower overpotential of 252 mV was observed compared to the LSV measurement using the Pt wire counter electrode (264 mV).

The promising performance of 2D-Ta_2_Se_2_C indicates that delaminating Ta_2_Se_2_C and exposing more catalytic active sites remarkably improve its performance for HER and is comparable to previously reported electrocatalysts for HER.^9,55−58^ For example, Thangasamy et al. exfoliated MoS_2_ powder using sonication at different dispersion media achieving an η_10_ of 570 to 720 mV.^55^ In another study, Thangasamy et al. used a solvothermal approach to produce a rose-like shape MoS_2_ nanostructure, resulting in an η_10_ of 330 mV.^55^ Chen et al. synthesized TaS_2_ by electrochemically exfoliating bulk TaS_2_ using an alternating voltage in an acidic electrolyte, with an η_10_ of 370 mV on GC.^56^

To further understand the HER kinetics and inherent activities of m-Fe_0.2_Ta_2_Se_2_C and 2D-Ta_2_Se_2_C samples for the HER, Tafel polarization curves were plotted using data from LSV curves (Figure 2b). The Tafel slope for 2D-Ta_2_Se_2_C in the presence of H_2_ gas in the system was calculated to be 91 mV·dec^–1^, indicating the HER kinetics is controlled by the Volmer step^57^ and much lower than m-Fe_0.2_Ta_2_Se_2_C (185 mV·dec^–1^). This indicates the higher intrinsic activity of 2D-Ta_2_Se_2_C due to the synergistic effect of the large, exposed surface area, the presence of iron as single atoms, and vacancies in the structure, which provide a more open structure. As reported in the literature for TMDs, vacancies enhance the catalytic activity in HERs by creating active sites and altering the electronic structure. Sulfur (S) and Selenium (Se) vacancies in 2D-TMDs lower hydrogen adsorption energy, making the surface more reactive and improving the efficiency of hydrogen adsorption and desorption.^58^ These vacancies also increase the number of available edge sites, which are crucial for efficient HER catalysis. Similarly, in TMCCs, tuning the vacancy concentration can potentially optimize the catalytic performance of 2D-TMCCs, making them promising candidates to replace noble metal catalysts in the HER. Understanding the role of Se vacancies in 2D-Ta_2_Se_2_C is essential for enhancing its catalytic efficiency in hydrogen evolution. Further investigation into how these vacancies affect the material’s electronic properties and catalytic activity will provide deeper insights into its performance and guide the development of more efficient catalysts for energy conversion applications.^58,59^

Then, CVs at different scan rates of 20, 40, 60, 80, 100, and 200 mV·s^–1^ were measured for 2D-Ta_2_Se_2_C (Figure 2c) with bubbling H_2_. H_2_ purging was performed to saturate the electrolyte, ensuring a consistent electrocatalytic performance. To confirm that this does not affect the accessibility to active sites, we measured the CVs with and without bubbling H_2_, and the results were nearly identical (Figure S9b). The CVs show almost the same capacitance, indicating that the low flow rate of H_2_ bubbling used in this experiment does not hinder the accessibility of the active sites. By extracting the values of the difference between anodic and cathodic current densities at 0.30 V corresponding to different scan rates, we obtained the plot in Figure 2d. Assuming no Faradaic reaction contribution, the slope of this line is equal to the double-layer capacitance (*C*_dl_).^60^ A *C*_dl_ of about 16.2 mF·cm^–2^ was measured, corresponding to a specific electrochemically active surface area (ECSA) of 17.61 m_ECSA_^2^·g_catalyst_^–1^ (details of the calculations and assumptions are provided in the Supporting Information). Compared to the theoretical specific surface area of 212.82 m^2^·g^–1^, the lower ECSA estimated here suggests incomplete utilization of Ta_2_Se_2_C or that the basal plane might not be the active site for HER. The ECSA and *C*_dl_ values are higher than what was reported for ultrathin Ti_3_C_2_T_*x*_ MXene (ECSA = 12.07 m_ECSA_^2^·g_catalyst_^–1^), MoS_2_ (2 m_ECSA_^2^·g_catalyst_^–1^),^55^ and TaSe_2_ nanobelts (13.12 m_ECSA_^2^·g_catalyst_^–1^),^61^ indicating the high effective surface area of the 2D-Ta_2_Se_2_C electrocatalyst. Table S8 compares the electrocatalytic performance of 2D-Ta_2_Se_2_C with various 2D-TMDs reported in the literature, demonstrating that 2D-Ta_2_Se_2_C exhibits a performance on par with some of the most active 2D-TMDs. Also using CVs at different scan rates, the capacitance versus potential graph was extracted (Figure S9c), demonstrating uniform capacitance behavior across the scan rates.

The chronoamperometric stability graph for D-Ta_2_Se_2_C in Figure S9d reveals promising electrochemical stability under continuous operation at room temperature under H_2_ bubbling at a constant applied potential of −0.3 V. The initial sharp decrease in current density observed in the first few hundred seconds likely represents the formation of a stable electrochemical double layer and the initial activation of surface sites. Following this, the current stabilizes, with a gradual increase, suggesting that the material undergoes further surface restructuring or stabilization of active sites, improving its conductivity and catalytic activity over time. Importantly, the absence of significant fluctuations or current decay throughout the 1500 s test indicates that 2D-Ta_2_Se_2_C exhibits durable electrochemical performance without apparent degradation. This stable behavior suggests that 2D-Ta_2_Se_2_C can maintain its activity over extended electrochemical cycles, making it a strong candidate for applications requiring sustained electrochemical activity, such as in energy conversion or storage technologies.

To compare the chemical stabilities of 2D-Ta_2_Se_2_C and 2D-TaSe_2_, the colloidal dispersion obtained after delamination was stored at room temperature for several days, and photographs were taken at different time intervals (Figure S11). The results showed that the 2D-TaSe_2_ solution began to change color within the first few hours, indicating lower stability compared to that of 2D-Ta_2_Se_2_C, which exhibited almost no color change even after several days.

The turnover frequency (TOF) versus potential graph obtained from the LSV results for 2D-Ta_2_Se_2_C in Figure S9f shows that the TOF increases as the applied overpotential increases from −0.2 V vs reversible hydrogen electrode (RHE) to more negative (−0.38 V vs RHE). This suggests that the catalytic activity improves significantly at more negative potentials, indicating an enhanced reaction kinetics in this region. The red plot is when we consider the Se sites at the edge to be active (0.4% of the total amount of Se), and the blue plot is when we consider the Se sites at the basal plane to be active (details of the calculations and assumptions are provided in the Supporting Information).

It is worth noting that, as discussed earlier, bulk phases such as TaC particles, which are not known to be effective catalysts for HER,^62^ coexisted with delaminated Ta_2_Se_2_C. Therefore, it is reasonable to predict that better performance can be achieved by further purifying the sample and synthesizing higher-purity materials. Another factor that might contribute to the electrocatalytic behavior of the studied materials is the presence of small amounts of lithium and iron in the sample. Iron is known to be an effective catalyst for HER, particularly when used as a single-atom catalyst.^63^ For instance, an overpotential as low as 9 mV and a favorable Tafel slope of 37.8 mV·dec^–1^ were reported for iron/graphdiyne.^64^ However, the iron residues from the solid-state synthesis step of bulk Ta_2_Se_2_C, which persisted in the materials after delamination, are not expected to lead to single-atom dispersion of iron on the surface of Ta_2_Se_2_C sheets. Instead, they likely form particles of iron and iron oxide, as observed through XPS analysis. Further studies are needed to understand the nature of iron and lithium in TMCCs and their effect on the electrochemical behavior of TMCCs.

### DFT Calculations

**Figure 3:**
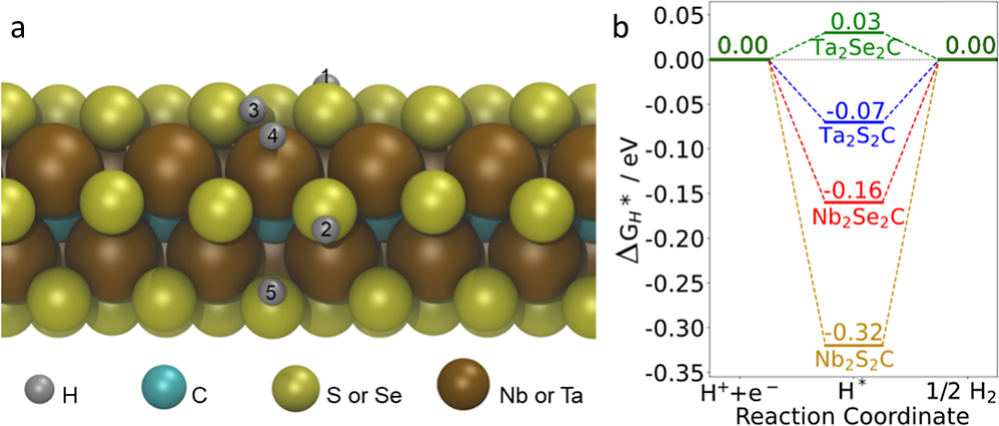
(a) Side view of H adsorption on different positions of a TMCC edge. (b) Δ*G*_H*_ of H(5) on different TMCC edges.

To gain atomistic insight into the reactivity trends and the likely active sites, H adsorption on various TMCCs (Ta_2_Se_2_C, Ta_2_S_2_C, Nb_2_Se_2_C, and Nb_2_S_2_C) was calculated by DFT. The TMCCs were modeled including an edge so that both edge and plane sites could be studied. One H atom was placed at various positions, and 5 relatively low-energy converged structures were found, as shown in Figure 3a. H(1) is on top of a surface S/Se, similar to adsorption on the basal plane of a 2D surface; H(3) is bridging an S/Se and a metal atom at the edge; H(4) is on top of a metal atom at the edge; H(2) and H(5) are binding to two different edge S/Se atoms. The calculated Δ*G*_H*_ values at these 5 positions for the four TMCCs are listed in Table S9. Among the 5 positions, H(5) always had the strongest adsorption, and its Δ*G*_H*_ was the closest to 0. This indicates that H(5), an S or Se edge site, is likely to be most active for HER. The Δ*G*_H*_ of H(5) on 4 TMCC edges are shown in Figure 3b, with an order of Ta_2_Se_2_C > Ta_2_S_2_C > Nb_2_Se_2_C > Nb_2_S_2_C. The results indicate that the TMCC with Nb has stronger adsorption than Ta, and S has stronger adsorption than Se. Ta_2_Se_2_C is the nearest to the zero level, which suggests it would have the highest catalytic performance for HER among the 4 materials. The Ta_2_Se_2_C sites have an H adsorption energy quite close to 0, which might be expected to lead to an overpotential close to 0. However, the H adsorption energy alone is not quantitatively accurate in predicting electrocatalytic performance, as the details of the mechanism and transition states and the effect of the complex electrocatalytic environment all play a role in determining the performance.^65^

To gain insight into how the C core may generally lead to conductivity in TMCCs, we calculated the projected density of states (PDOS) for Ta_2_Se_2_C and 1H–TaSe_2_ (Figure S12). 1H–TaSe_2_ is itself metallic, and our calculations correctly capture this (see Figure S12b) as do previous calculations.^66^ Our calculations also correctly predict Ta_2_Se_2_C to be metallic (see Figure S12a). In Ta_2_Se_2_C, we found that the C core did not directly contribute to a large number of states in the region around the Fermi energy but instead modified the PDOS on the other two elements. Specifically, TaSe_2_ shows more isolated peaks with gaps between them, while for Ta_2_Se_2_C, there is more overlap between peaks and fewer gaps. This smoother behavior with few gaps was also seen in previous PDOS calculations of TMCCs.^67^ This reduction in the number of gaps would be expected to generally promote metallic behavior, consistent with the more metallic nature of TMCCs as compared to TMDs.

## Conclusions

In summary, to obtain efficient electrocatalysts containing core carbon layers of MXenes which provide high electrical conductivity as well as stability and the surface layer of TMDs which provides high electrochemical activity, we produced multilayer Ta_2_Se_2_C for the first time through a simple scalable and rapid solid-state thermal process under atmospheric pressure. Ta, TaC, and FeSe were mixed, pressed, and heated for 4 h at 1200 °C. Most of the iron in the secondary phase and the iron between the layers were removed by etching in 3 M H_2_SO_4_. Electrochemical Li-intercalation followed by sonication in DI water exfoliation led to the synthesis of Ta_2_Se_2_C mono-/few-layer sheets. Comparing measured and theoretically calculated XRD patterns confirmed the formation of the hexagonal Ta_2_Se_2_C structure (*P*3̅*m*1 space group). Microscopy techniques illustrated the layered hexagonal structure of the multilayer sample and few-layer sheets for the delaminated sample with about 4 nm thickness. STEM–EELS quantified a Ta/Se ratio of almost 2.0:1.8, suggesting Se vacancies in the structure most likely formed during the purification and liquid exfoliation procedures.

We investigated the electrocatalytic performance of the drop-casted sample on a GC electrode which illustrated an improved HER electrocatalytic performance of 2D-Ta_2_Se_2_C (η_10_ = 264 mV), compared to the multilayered Ta_2_Se_2_C (η_10_ = 678 mV), due to a synergistic effect of inherent catalytic performance of the material possibly as a result of high electrical conductivity and catalytic activity, and an ECSA of about 17.61 m_ECSA_^2^ g_catalyst_^–1^. Using DFT calculations to analyze reactivity trends and active sites for H adsorption on various TMCCs, we identified five potential active sites, with the strongest adsorption occurring at the binding edge S/Se atoms, exhibiting an adsorption free energy of 0.03 eV. This suggests that these sites are the most active for HER. Comparison among different TMCCs revealed Ta_2_Se_2_C as the top performer for HER catalysis, with the order of catalytic activity as Ta_2_Se_2_C > Ta_2_S_2_C > Nb_2_Se_2_C > Nb_2_S_2_C.

Our study proposes a scalable synthesis method for multilayer and 2D-TMCCs or TMCC nanoparticles, facilitating the design of efficient electrocatalysts for the HER and likely other energy storage applications.

## Experimental Section

### Synthesis of Multilayer Fe_*x*_Ta_2_Se_2_C

A simple solid-state method was used to synthesize Fe_*x*_Ta_2_Se_2_C. In a typical procedure, Ta (Alfa Aesar, <44 μm, 99.9% purity), TaC (Alfa Aesar, <44 μm, 99.9% purity), and FeSe (Thermo Scientific, <420 μm, 99.9% purity) powders were weighed in an argon (Ar)-filled glovebox under an ultrahigh-purity (UHP) Ar atmosphere. Powders in the molar ratio of Ta/TaC/FeSe = 1.0:1.0:2.0 (each batch was 10 g) were placed in a 30 mL high-density polyethylene (HDPE) jar containing 20 yttrium-stabilized zirconia balls of 5 mm diameter each. The HDPE jar was closed and sealed using parafilm and removed from the glovebox for mixing. A Turbula T2F mixer at ≈56 rpm for 3 h was used to mix the powders. Then, the jar was transferred to the glovebox again, and the powders were pressed into a 1″ diameter pellet under 350 bar, put in an alumina crucible, and transferred in a sealed container to a tube furnace that was in a well-ventilated chemical fume hood. The sample was heated to 1200 °C at a heating rate of 10 °C·min^–1^ and held at 1200 °C for 4 h, and then the furnace was allowed to cool to room temperature. The heating and cooling treatments were carried out under a continuous Ar flow of about 3 L·min^–1^. Other synthesis conditions for Fe_*x*_Ta_2_Se_2_C, including different precursor compositions, synthesis temperatures, and times, were explored and are listed in the Supporting Information (Table S1). The method used to synthesize Fe_*x*_TaSe_2_ is explained in Supporting Information and the corresponding XRD plot is provided in Figure S2.

To remove excessive iron, the sample was ground to −325 mesh (<44 μm), and 1 g of Fe_*x*_Ta_2_Se_2_C powder was gradually added to 20 mL of 3 M H_2_SO_4_ and stirred for 24 h at room temperature. The mixture was sonicated every few hours for 1 min to facilitate acid penetration into the powder particles with a total sonication duration of 5 min. Then, the acid was replenished, and the procedure was repeated for another 24 h. Then, the solution was centrifuged at 3500 rpm for 3 min, the acid was decanted, and settled powders were washed with DI water to pH ≈ 7 and vacuum filtered on a Whatman filter paper grade 2 overnight.

### Synthesis of 2D-Ta_2_Se_2_C

To exfoliate the layers of Ta_2_Se_2_C and obtain single- or few-layer sheets, we used the electrochemical Li-intercalation technique (voltage profile in Figure S3) followed by exfoliation via sonication in DI water. About 120 mg of the multilayer powder was pressed to a free-standing 1″ diameter wafer under 500 bar. The wafer was transferred to an Ar-filled glovebox (O_2_ < 0.1 ppm, H_2_O < 0.1 ppm). The schematic configuration of the electrochemical cell (EL CELL) is shown in Scheme S1. A stainless-steel disk of 14 mm diameter was used as the bottom-part current collector. Both sides of a Li wafer were brushed to get a shiny surface and pressed on the stainless-steel disk to attach to it and get a mirror-like surface. A glass microfiber filter (Whatman CAT no. 1823–047 GF/D, 2.7 μm) was placed on the Li wafer, and 100 μL of 1 M LiPF_6_ in ethylene carbonate and ethyl methyl carbonate (EMC) (EC/EMC equaled 3:7 by volume) was dropped all over the separator as an electrolyte. Then, the free-standing wafer and a copper foil of 1″ diameter were added as the working electrode and current collector, respectively. The cell was sealed tightly, removed from the glovebox and connected to a potentiostat–galvanostat (VMP3, BioLogic). Galvanostatic cycling with potential limitation (GCLP) was conducted with a low discharge rate of 5 mA·g^–1^ started from the OCP to a cutoff potential of 0.1 V vs Li/Li^+^. During discharge, Li intercalates between the layers of Ta_2_Se_2_C. Figure S3 shows the voltage profile for a 120 mg wafer. After reaching the cutoff potential, the EL CELL was disconnected and transferred to the glovebox, and the working electrode was removed from the EL CELL, soaked in diethyl carbonate (DEC) to rinse off any remaining salts, removed from DEC, cleaned gently with Kimwipe, and then put in a 50 mL centrifuge tube. The tube cap was tightened before removal of the tube from the glovebox and transferred to an ultrasonic bath. Sonication at 37 MHz was started while adding 50 mL of DI water to the tube. A 5 min sonication followed by one h centrifugation at 5000 rpm was conducted as the first cycle. The supernatant was separated and discarded, 50 mL of DI water was added to the sediment and redispersed, and the second cycle was conducted with 5 min sonication and a 30 min centrifugation at 3500 rpm. The supernatant was collected in a bottle. 50 mL of DI water was added to the sediment, and the previous cycle of sonication and centrifugation was repeated until a clear liquid was obtained after centrifugation. Finally, the collected liquid was filtered or freeze-dried. The material on the filter formed a free-standing paper, and the material from the freeze drier formed an aerogel. The schematic procedure used here for synthesizing 2D-Ta_2_Se_2_C is provided in Scheme 1.

### Characterization of Multilayer and Delaminated Ta_2_Se_2_C

A Cu K_α_ X-ray diffractometer Rigaku D/Max-2200 was used to collect XRD patterns at a 2θ step size of 0.02° and a sweep rate of 1°·min^–1^ at operation conditions of 40 kV and 40 mA. SEM images were captured using a Hitachi S-4800 with an acceleration voltage of 3 kV. For elemental analysis, we used an SEM Hitachi S-3400 equipped with an energy-dispersive X-ray spectroscopy detector (EDS, Oxford, UK) at an accelerating voltage of 30 kV connected to INCA software. Elemental concentrations were determined using the Thermo Element2 high-resolution ICPMS at Tulane University’s TINI department. Linear calibration curves were established for each element with a laboratory blank subtracted. The sample solution was prepared by dissolving 2D-Ta_2_Se_2_C in an aqua regia solution with hydrofluoric acid addition. Results were reported as parts per billion after appropriate dilution using 2% nitric acid. A solution of 0.6 g·L^–1^ was prepared, and UV–vis absorption spectra were recorded using a UV-1700 PharmaSpec UV–vis Spectrometer (Shimadzu Corporation), equipped with UVProbe software (Version 2.35) for data acquisition and analysis.

TEM images were captured by an FEI TECNAI G2-F30 transmission electron microscope at an accelerating voltage of 200 kV. To get a better understanding of the elemental composition and the atomic-scale configuration of the structure, EELS together with STEM imaging using a HAADF detector was carried out using the Linköping double C_s_-corrected FEI Titan^3^ 60–300 microscope operated at 300 kV and the embedded GIF Quantum ERS. The probe maintained a convergence angle of 21.5 mrad throughout the imaging and EELS acquisition. EELS spectrum images (SIs) were acquired for 5 min using 0.1 nA beam current, 0.25 eV/channel energy dispersion, 0.2 s pixel dwell time, and a 55 mrad collection semiangle. To prepare the sample, a multilayer powder was mixed with distilled water to make a liquid suspension. Then the liquid suspension was sonicated for 2 min to break the large Ta_2_Se_2_C multilayers into small particles to avoid charging issues. A few drops from the liquid suspension were then dropped onto the copper lacey carbon grid (300 mesh) and allowed to dry for a few minutes. Then, the grid was loaded into a standard double-tilt holder and introduced into the microscope.

For surface chemistry investigation, we used X-ray photoelectron spectroscopy (XPS, Thermo Fisher, USA) with an Al K_α_ X-ray source at a 200 eV pass energy, a step of 1 eV, and a spot size of 400 μm. The XPS sample was prepared by drop-casting a diluted slurry of the aerogel sample in a nonaqueous liquid on a copper substrate, dried in a vacuum under an Ar environment, and exposed to ion-irradiation for charge neutralization, followed by a 5 min Ar^+^ sputtering to remove the surface and clean any probable contaminants during sample preparation. CasaXPS software was used for the XPS data analysis and peak fitting.

The samples for transmission electron microscopy (TEM) studies were prepared from the fresh supernatant after delamination and centrifugation, and just extra ethanol was added to dilute the supernatant. The colloidal dispersion of 2D-Ta_2_Se_2_C was then drop-cast onto a TEM copper grid. A multimode atomic force microscope (Bruker Dimension ICON, USA) was used to image the single sheets and measure their thickness. The atomic force microscopy (AFM) sample was prepared by spray-coating DI water-diluted 2D-Ta_2_Se_2_C colloidal dispersion after exfoliation onto an Ar plasma-cleaned Si substrate. The AFM tip (PPP-NCHR-10, NANOSENSORS) operated under tapping mode to collect the AFM image.

The electrical conductivity of the samples was measured using a ST2253 digital four-probe resistivity meter.

### Performance for HER

The electrochemical activities of multilayer and delaminated Ta_2_Se_2_C were investigated using standard three-electrode cells. Electrodes of drop-cast samples on a rotating disk of glassy carbon (RDGC) were used as the working electrodes. Platinum wire and Hg/HgSO_4_ in saturated KCl were used as counter and reference electrodes, respectively. An aqueous 0.5 M H_2_SO_4_ solution was used as the electrolyte. Before electrochemical measurements were started, the electrolyte was bubbled with H_2_ or Ar gas for at least 30 min and kept bubbling during measurements. The cell was connected to a BioLogic Potentiostat-Galvanostat equipped with EC-lab software.

To prepare the working electrodes, 10 mg of each sample was added to a solution containing 100 μL of DI water, 90 μL of ethanol, and 10 μL of Nafion solution (alcohol-based, 1000 EW at 5 wt %, ION POWER). The slurry was sonicated for 15 min at 25 °C. Then, 3.3 μL of the slurry was dropped on the surface of a mirror-like polished RDGC with a 3 mm diameter and dried under ambient conditions for about 30 min (loading ∼2.3 mg·cm^–2^).

To investigate the electrochemical performance, first, the OCP of the cell was measured, and 20 CVs were performed with a potential window of OCP ±50 mV at a scan rate of 20 mV·s^–1^. LSV was conducted at a scan rate of 5 mV·s^–1^ in a potential window of 0 to −0.8 V vs RHE. The PEIS was performed at a range of 100 kHz to 100 mHz with a sinus amplitude of 10 mV to appraise the reaction kinetics and to measure the charge transfer resistance (*R*_ct_) at the electrode/electrolyte interface,^68^ and all the potential values were *iR*-corrected and reported versus RHE according to *R*_ct_ obtained from the PEIS measurements. The current values were converted to the geometric current density. The Tafel polarization slope was calculated from the LSV data. CVs at different scan rates were recorded, and from that, the *C*_dl_ of each material was extracted in the nonfaradaic region, and from that, the ECSA was calculated.^69^

### Computational Method

All calculations in this work were performed applying DFT with the Perdew, Burke, and Ernzerhof generalized gradient functional (GGA-PBE)^70^ with the Tkatchenko–Scheffler method^71^ for van der Waals interactions. A 7 × 1 × 1 *k*-point mesh was used with a 400 eV cutoff energy. All calculations were done using the Vienna Ab initio Simulation Package (VASP),^72^ and structures were visualized using Visual Molecular Dynamics.^72^

In this work, a 3 × 3 unit cell was used containing nine M_2_S_2_C or M_2_Se_2_C formula units (M = Nb or Ta). Roughly 12 Å of vacuum was inserted in the *y*-direction to form an edge and the *z*-direction to separate the layers. All atoms were relaxed during optimization. The Gibbs free energy (300 K) of hydrogen adsorption was defined as
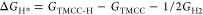
where *G*_TMCC-H_ is the free energy of a TMCC with an adsorbed H atom, *G*_TMCC_ is the free energy of the clean TMCC, and *G*_H2_ is the free energy of the hydrogen molecule in the gas phase. The free energy *G* was calculated as
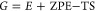
where *E* is the electronic energy, ZPE is the zero-point energy, *T* is the temperature, and *S* is the entropy.

## Supporting Information Available

Further details on the synthesis, characterization, and additional electrochemical results for Ta_2_Se_2_C and TaSe_2_ and calculations for ECSA, theoretical specific surface area, and TOF for 2D Ta_2_Se_2_C (PDF)
