# Supplementary material for: Two-Dimensional Tantalum Carbo-Selenide for Hydrogen Evolution
Source: ACS Nano. 2025 Jan 16;19(3):3185–96. doi: 10.1021/acsnano.4c09903 (PMC11781020; doi:10.1021/acsnano.4c09903)
Supplement: Supplementary file 1 — nn4c09903_si_001.pdf [file nn4c09903_si_001.pdf]

Supporting information

Two-Dimensional Tantalum Carbo-Selenide for Hydrogen Evolution

*Elham Loni,[†] Ahmad Majed,[†] Shengjie Zhang,[‡] Hari H. S. Thangavelu,[§] Chaochao Dun,[//] Anika Tabassum,[†] Karamullah Eisawi,[†] Jeffrey J. Urban,[//] Per O. Å. Persson,[§] Matthew M. Montemore,[‡] and Michael Naguib[†] **

[†]Department of Physics and Engineering Physics, Tulane University, New Orleans, LA 70118, USA.

[‡]Department of Chemical and Biomolecular Engineering, Tulane University, New Orleans, LA 70118, USA.

[§]Department of Physics, Chemistry and Biology, Linköping University, SE-581 83 Linköping, Sweden.

[//]The Molecular Foundry, Lawrence Berkeley National Laboratory, Berkeley, CA 94720, USA.

Table S1: Different conditions for the synthesis of $Fe_xTa_2Se_2C$

Precursors	Temperature (°C)	Time (h)	Scan rate (°C·min^{-1})	Details
1Ta+1TaC+2FeSe	1300	4	10	-
1Ta+1TaC+2FeSe	1200	4	10	-
1.2Ta+0.8TaC+2FeSe	1200	4	10	-
1Ta+1TaC+2FeSe	1200	8	10	-
0.8Ta+1.2TaC+2FeSe	1200	4	10	-
1Ta+1TaC+2FeSe	1250	4	10	-
1Ta+1TaC+2FeSe	1150	4	10	-
1Ta+1TaC+2FeSe	1300	4	10	Reheated
1Ta+1TaC+2FeSe	1200	4	10	Reheated

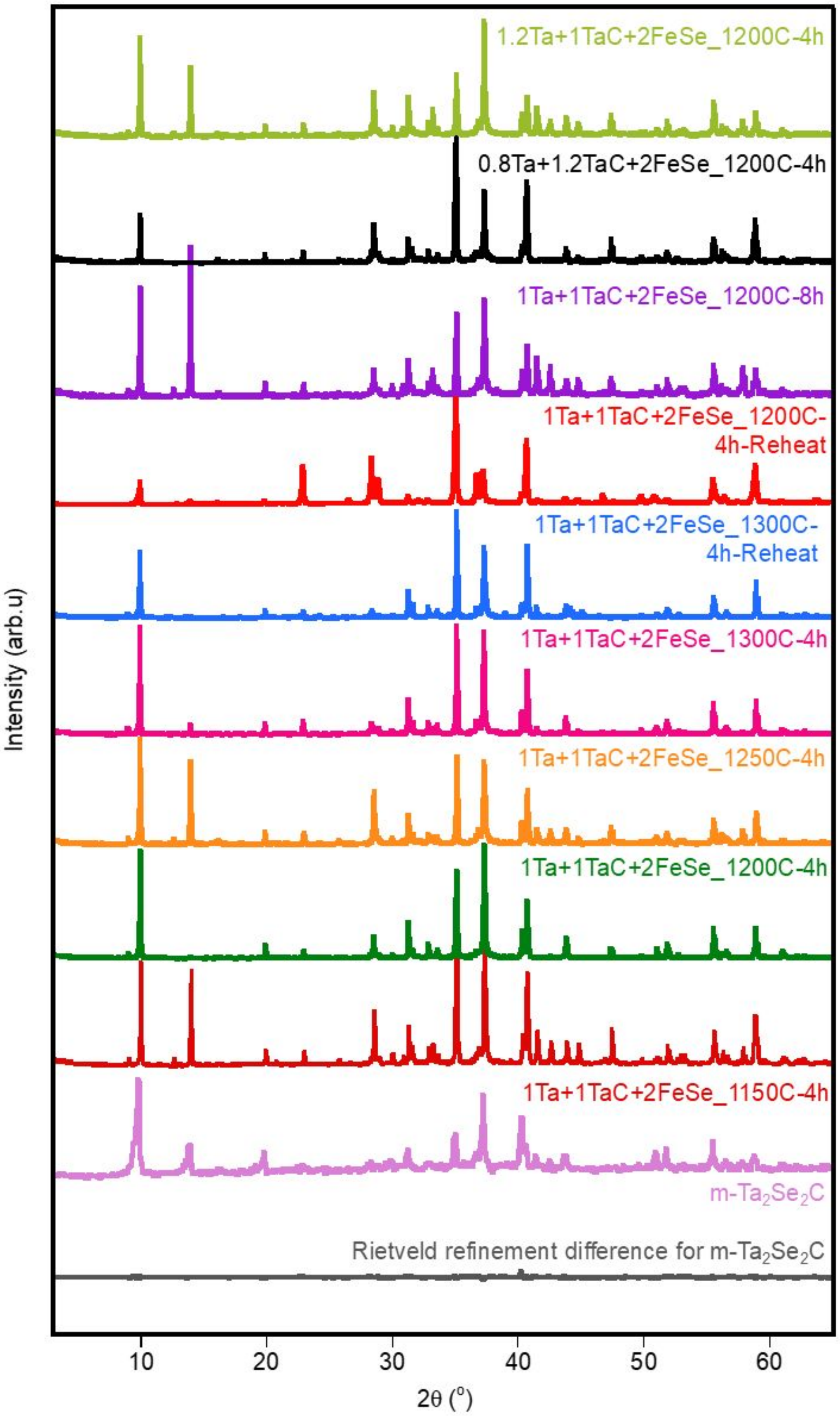

Figure S1: XRD patterns of all samples, demonstrate the influence of heating temperature, duration, and precursor molar ratios. The gray curve depicts the residual difference between the Rietveld refinement and the experimental

XRD data for the multilayer sample, derived from etching the "1Ta+1TaC+2FeSe_1200C-4h" sample (shown in green).

Rietveld analysis of the sample obtained from solid-state synthesis process:

Phase name	$Fe_xTa_2Se_2C$
R-Bragg	4.574
Spacegroup	P-3m1
Scale	2.30799e-005
Cell Mass	3158.877
Cell Volume (Å^3)	84.70981
Wt% - Rietveld	52.586
Lattice parameters	
a (Å)	3.3068378
c (Å)	8.9449370

Site	Np	x	y	z	Atom	Occ	Beq
Ta0	6	0.66667	0.33333	0.86741	Ta	1	1
Ta1	6	0.33333	0.66667	0.13259	Ta	1	1
C2	1	0.00000	0.00000	0.00000	C	1	1
Se3	6	0.33333	0.66667	0.68572	Se	1	1
Se4	6	0.66667	0.33333	0.31428	Se	1	1
Fe5	1	0.00000	0.00000	0.50000	Fe	0.5009	1

Rietveld analysis of the sample obtained from etching:

Phase name	Ta_2Se_2C
R-Bragg	4.481
Spacegroup	P-3m1
Scale	3.74229e-004
Cell Mass	3130.905
Cell Volume (Å^3)	85.13885
Wt% - Rietveld	93.078
Lattice parameters:	
a (Å)	3.3126482
c (Å)	8.9587311

Site	Np	x	y	z	Atom	Occ	Beq
Ta0	6	0.66667	0.33333	0.86741	Ta	1	1
Ta1	6	0.33333	0.66667	0.13259	Ta	1	1
C2	1	0.00000	0.00000	0.00000	C	1	1
Se3	6	0.33333	0.66667	0.68572	Se	1	1
Se4	6	0.66667	0.33333	0.31428	Se	1	1

Table S2: XRD peak positions of the etched sample

Experimental 2θ	Theoretical 2θ	*h k l*	*d* (Å)
9.77	9.86	0 0 1	8.93
19.79	19.78	0 0 2	4.465
29.88	29.87	0 0 3	2.977
31.17	31.37	0 1 0	2.849
32.81	32.97	1 0 1	2.714
37.17	37.17	0 1 2	2.401
40.26	40.18	0 0 4	2.233
43.77	43.7	0 1 3	2.058
51.77	51.7	0 1 4	1.757

55.47	55.42	1 1 0	1.645
56.47	56.44	1 1 1	1.618
59.48	59.44	1 1 2	1.544
61.07	61.02	0 1 5	1.513
62.07	62.04	0 0 6	1.488

Synthesis of 2D-$TaSe_2$

The same solid-state method as $Fe_xTa_2Se_2C$ was used to synthesize Fe_xTaSe_2 with a Ta : FeSe = 1.0 : 2.0 (each batch was 10 g), followed by the same etching procedure, i.e., 48 h in 3 M H_2SO_4.

Then we used the electrochemical Li-intercalation technique followed by exfoliation via sonication in DI water.

Table S3: EDS results for Ta_2Se_2C and $TaSe_2$

Sample	Fe:Ta Molar ratio	Se:Ta Molar ratio
$Fe_xTa_2Se_2C$	1.00	1.00
Etched $Fe_xTa_2Se_2C$	0.09	0.62
2D-Ta_2Se_2C	0.25	0.80
Fe_xTaSe_2	2.00	2:00
m-$TaSe_2$	0.25	1.81
2D-$TaSe_2$	0.14	1.49

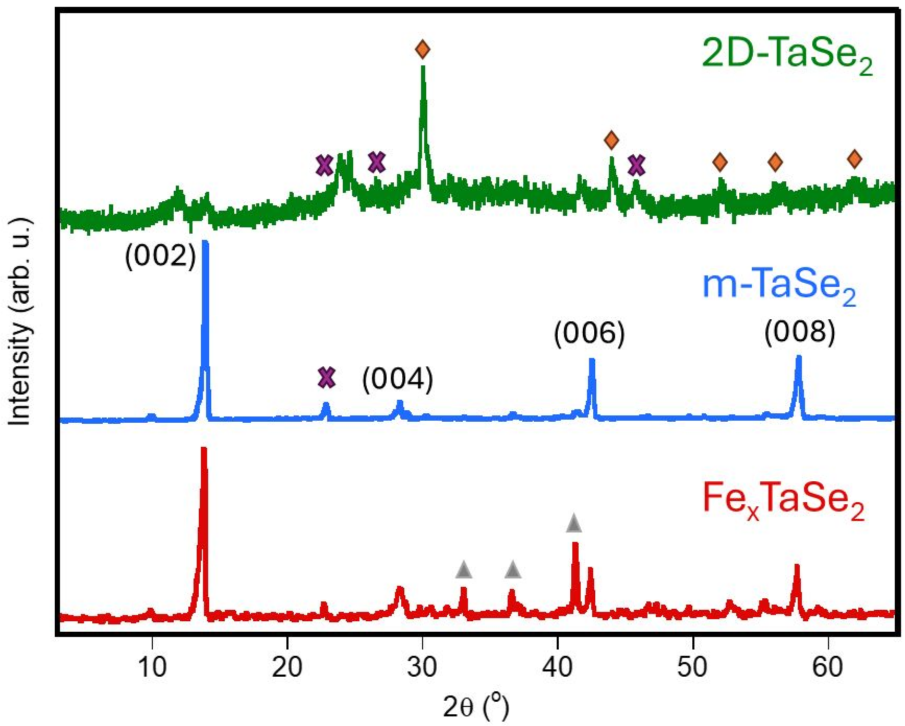

Figure S2: XRD pattern of Fe_xTaSe_2, m-$TaSe_2$ (PDF#21-1200), and 2D-$TaSe_2$ (X: Ta_2O_5; PDF#18-1304, ♦: Fe_3O_4; PDF# 00-065-3107, ▲: TaFe; PDF#21-0433)

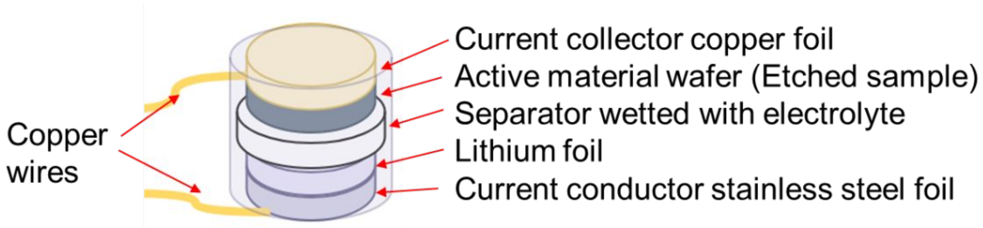

Scheme S1: Electrochemical cell schematic illustrating the cell components.

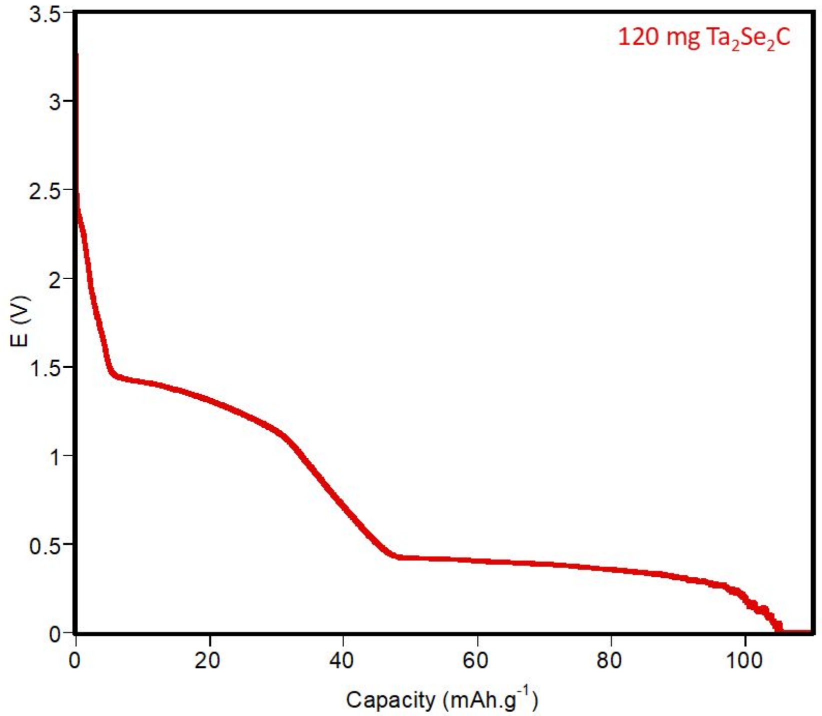

Figure S3: Voltage profile (potential versus specific capacity) for the lithiation of a 120 mg m-$Fe_{0.2}Ta_2Se_2C$ wafer.

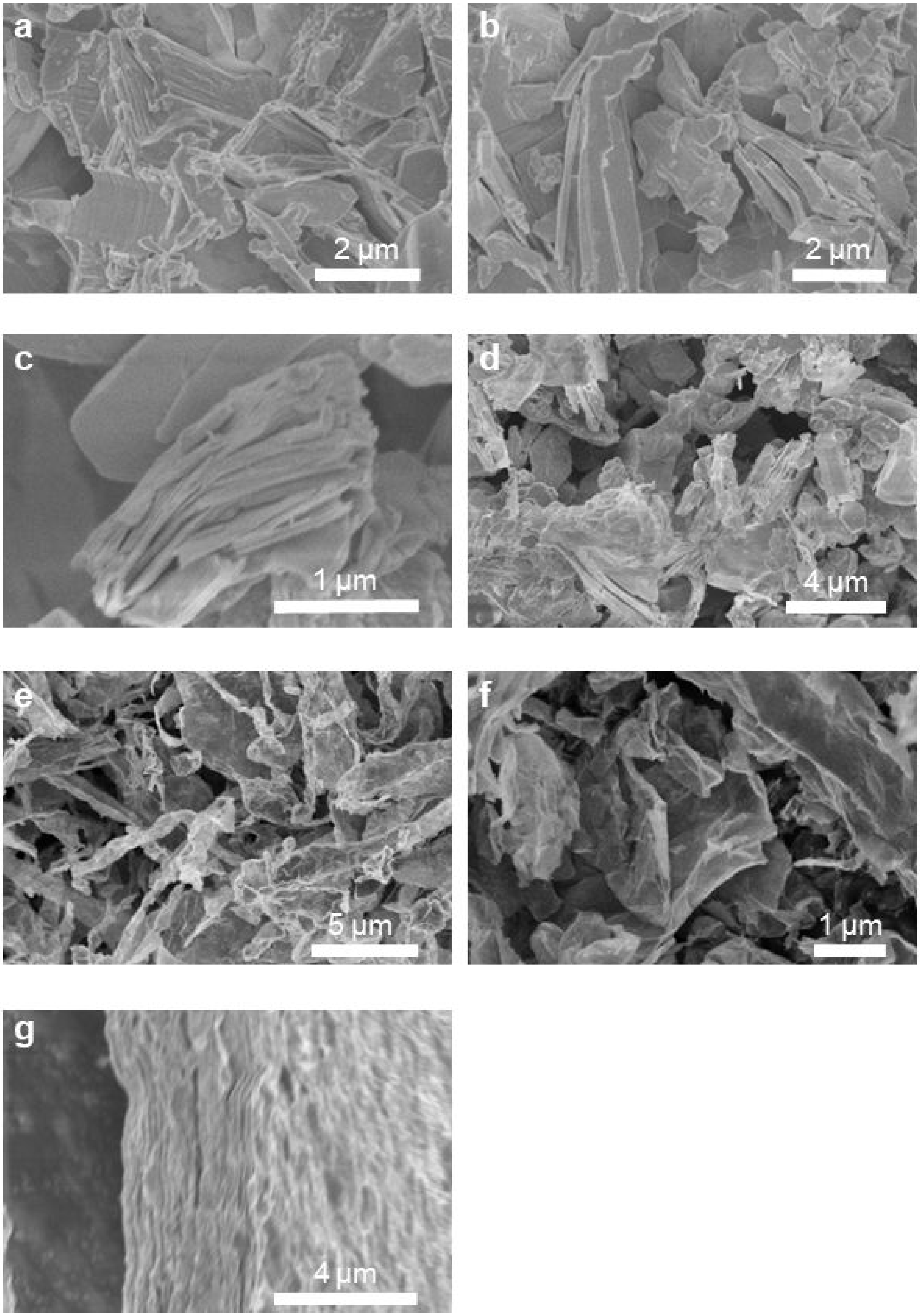

Figure S4: SEM images of a and b) as-synthesized, c and d) etched sample. e and f) 2D- Ta_2Se_2C survey for 2D-Ta_2Se_2C aerogel. g) SEM image of cross-section of Ta_2Se_2C free-standing paper.

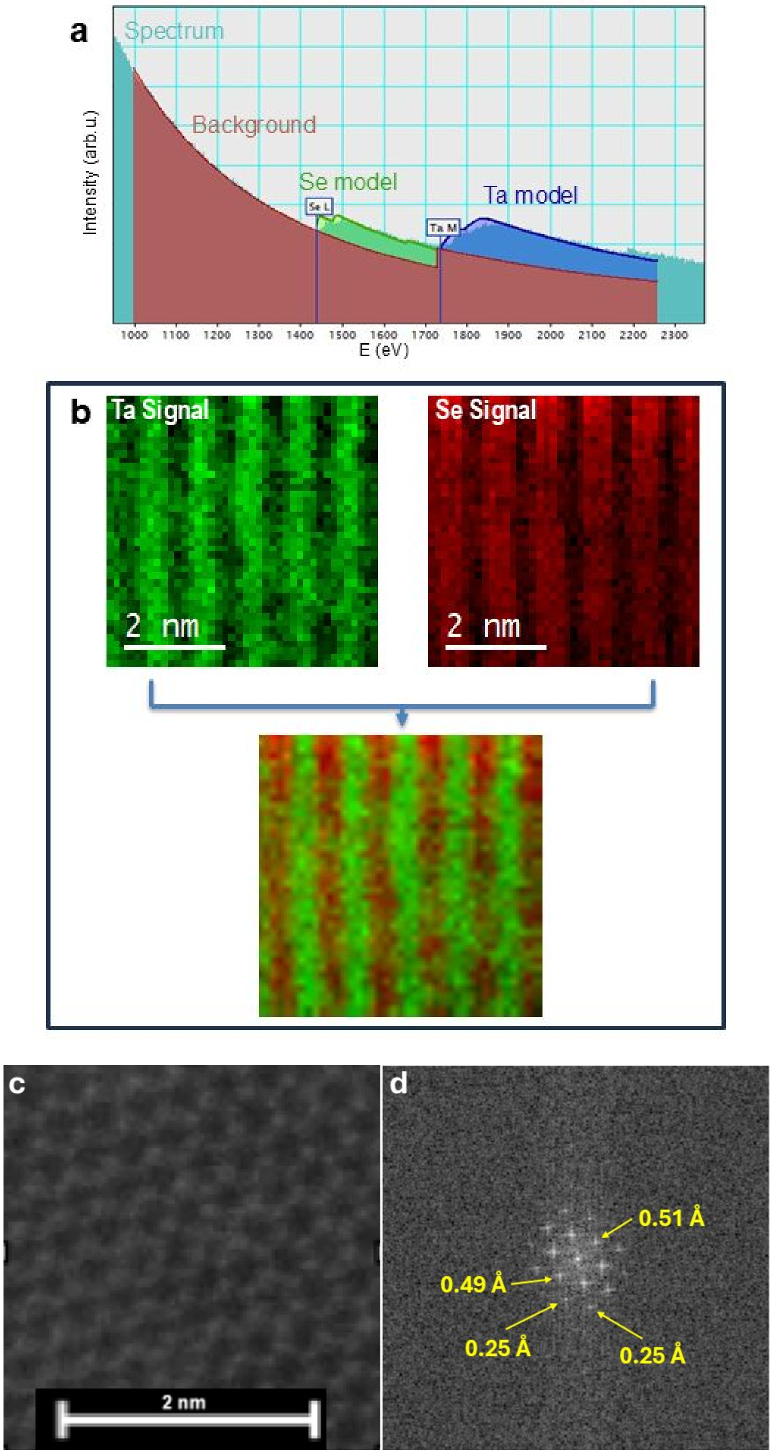

Figure S5: a) EELS EDS spectra of the m-$Fe_{0.2}Ta_2Se_2C$ particle corresponding to Figure 2g, and b) individual and mixed elemental maps for Ta (green) and Se (red), c) STEM overview for a single sheet of 2D-Ta_2Se_2C zoomed in, and d) extracted FFT pattern and calculated distance of atoms from the center spot.

Table S4: Composition results of EELS analysis

Spectrum	Element	Shell	Signal (e-)	Composition (At. %)	X-section (barns)	X-section Model
S1	Se	L	$5.1\times10^5 \pm 1.9\times10^3$	47.6	1795.9 ± 179.6	Hartree-Slater
	Ta	M	$1.8\times10^6 \pm 2.4\times10^3$	52.4	3567.6 ± 713.5	Hartree-Slater
S2	Se	L	$7.0\times10^5 \pm 1.8\times10^3$	43	1795.9 ± 179.6	Hartree-Slater
	Ta	M	$1.8\times10^6 \pm 2.3\times10^3$	57	3567.6 ± 713.5	Hartree-Slater

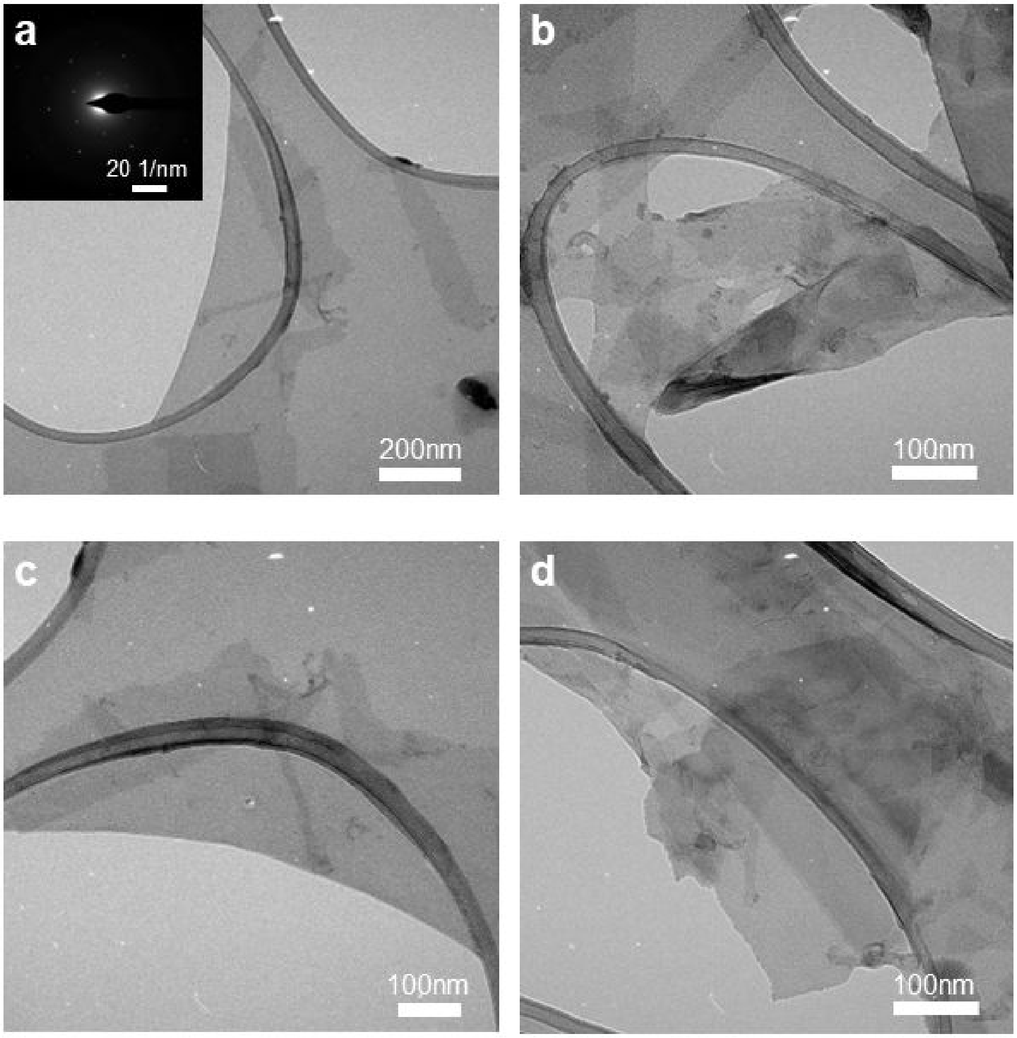

Figure S6: TEM images from 2D-Ta_2Se_2C. Inset: SAED pattern.

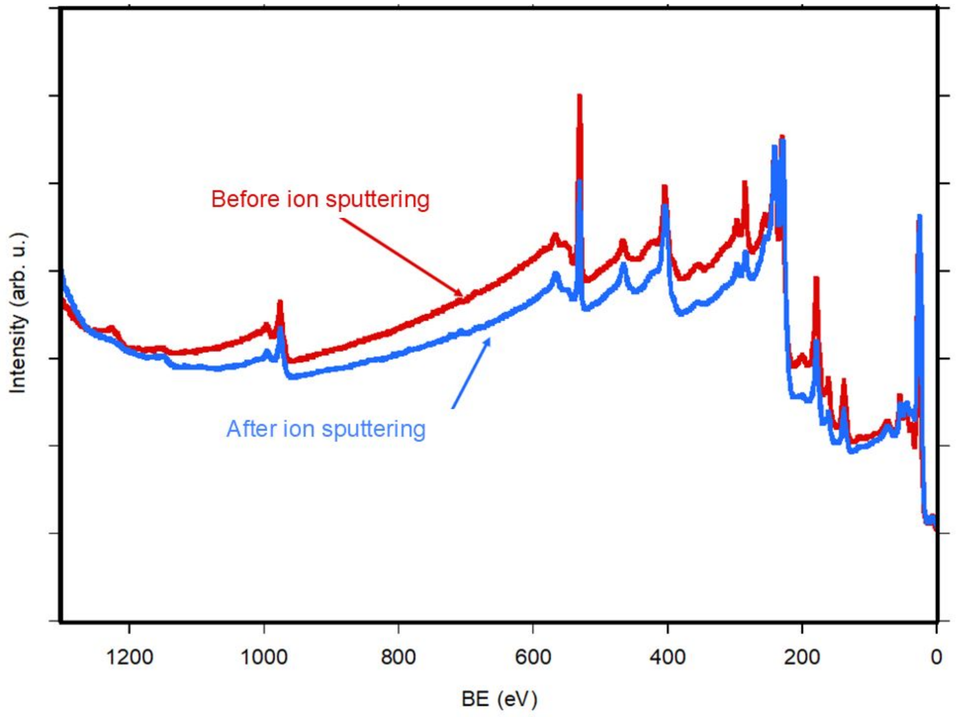

Figure S7: XPS survey for aerogel 2D- Ta_2Se_2C before and after ion sputtering.

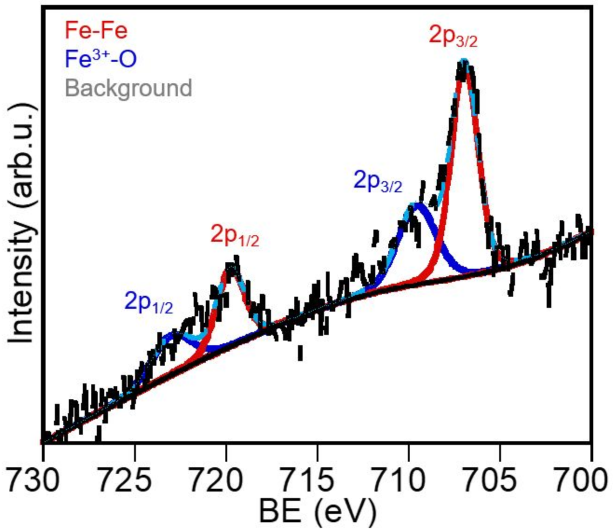

Figure S8: High-resolution XPS spectra for Fe 2p region.

Table S5: XPS peak fitting for d-Ta_2Se_2C: after 1 sputtering – calibrated midsection.

Elements	B.E. (eV)	FWHM (eV)	Fraction (%)	Assigned to	Mole%	Ref. (eV)
Ta $4f_{7/2}$ ($4f_{5/2}$)	22.6 (24.5)	1.7 (1.7)	56%	C-Ta-Se	7.5	23.4 Ta_2C[1] 22.8 TaS_2[2]
	23.2 (25.1)	1.3 (1.1)	5%	Se-Ta-Se	0.67	$TaSe_2$[3]
	24.2 (26.1)	1.9 (2.0)	11%	Ta^{+4}-O	1.47	
	26.4 (28.3)	1.8 (1.8)	28%	Ta^{+5}-O	3.74	
Se $3dp_{5/2}$ ($3d_{3/2}$)	53.4 (54.3)	1.2 (1.8)	63%	C-Ta-Se	6.76	
	54.1 (54.9)	0.8 (1.2)	14%	Se-Ta-Se	1.5	$TaSe_2$[4]
	54.9 (55.8)	1.2 (1.8)	23%	$Se-O_x$	2.4	
C 1s	282.6	1.9	52%	C-Ta-Se	14	
	284.4	1.7	26%	C-C/C-H		284.8[5]
	285.9	1.7	14%	C-OH/C-O-C	7.06	286.3[6]
	287.4	1.7	5%	C=O	3.68	287.6[6]
	288.4	1.7	3%	O-C=O	0.61	288.8[6]
O 1s	530.9	1.8	86%	$TM(OH)_x$	40.24	531.1[5]
	532.6	1.9	11%	O-C/$Se-O_x$	5.15	532.4[7]
	534.3	2	3%	$H_2O^{adv.}$	1.4	534.7[8]
Fe $2p_{3/2}$ ($2p_{1/2}$)	706.9 (719.7)	1.8	65%	Fe-Fe	1.78	706.6[9]
	709.5 (722.7)	2	35%	Fe^{3+}-O	0.96	709.8[9]

Table S6: ICPMS results for 2D-Ta_2Se_2C

Element	Concentration (ppb)	Normalized to 1 mole Ta
Li	5	0.38
Fe	13	0.12
Ta	342	1.00

Table S7: Electrical conductivity values for multilayer and delaminated Ta_2Se_2C and $TaSe_2$

Sample	m-$Fe_{0.2}Ta_2Se_2C$	2D-Ta_2Se_2C	m-$TaSe_2$	2D-$TaSe_2$
Electrical conductivity ($S \cdot cm^{-1}$)	2.75 ± 0.30	0.46 ± 0.02	2.71 ± 0.47	0.41 ± 0.03

Experimental ECSA calculations for Ta_2Se_2C:

The capacitance current density is proportional to the scan rate (ν) according to the following formula:

$i_c = C_{dl} \times \nu$

where, i_c is capacitance current density, C_{dl} is double-layer capacitance and ν is scan rate. Therefore, the slope of the curve i_c versus ν is equal to C_{dl}. C_{dl} was found to be 16.2 $mF \cdot cm^{-2}$.

Then, to calculate ECSA we used the following equation:
ECSA = C_{dl} / C_s ; where C_s is specific capacitance of the electrode and was reported to be in the range of 0.02–0.06 $mF \cdot cm^{-2}$ for a flat surface in H_2SO_4 electrolyte.[10-12] Thus, here we will assume an average value of 0.04 $mF \cdot cm^{-2}$.
Therefore, ECSA = 16.2 / 0.04 = 405 $cm^2_{ECASA}/cm^2_{geometric}$. Since loading of electrode ~ 0.0023 $g_{catalyst} \cdot cm^{-2}_{geometric}$, then the specific ECSA = 405/0.0023 = 176086.96 $cm^2_{ECSA} . g^{-1}_{catalyst}$ = 17.61 $m^2_{ECSA} . g^{-1}_{catalyst}$.

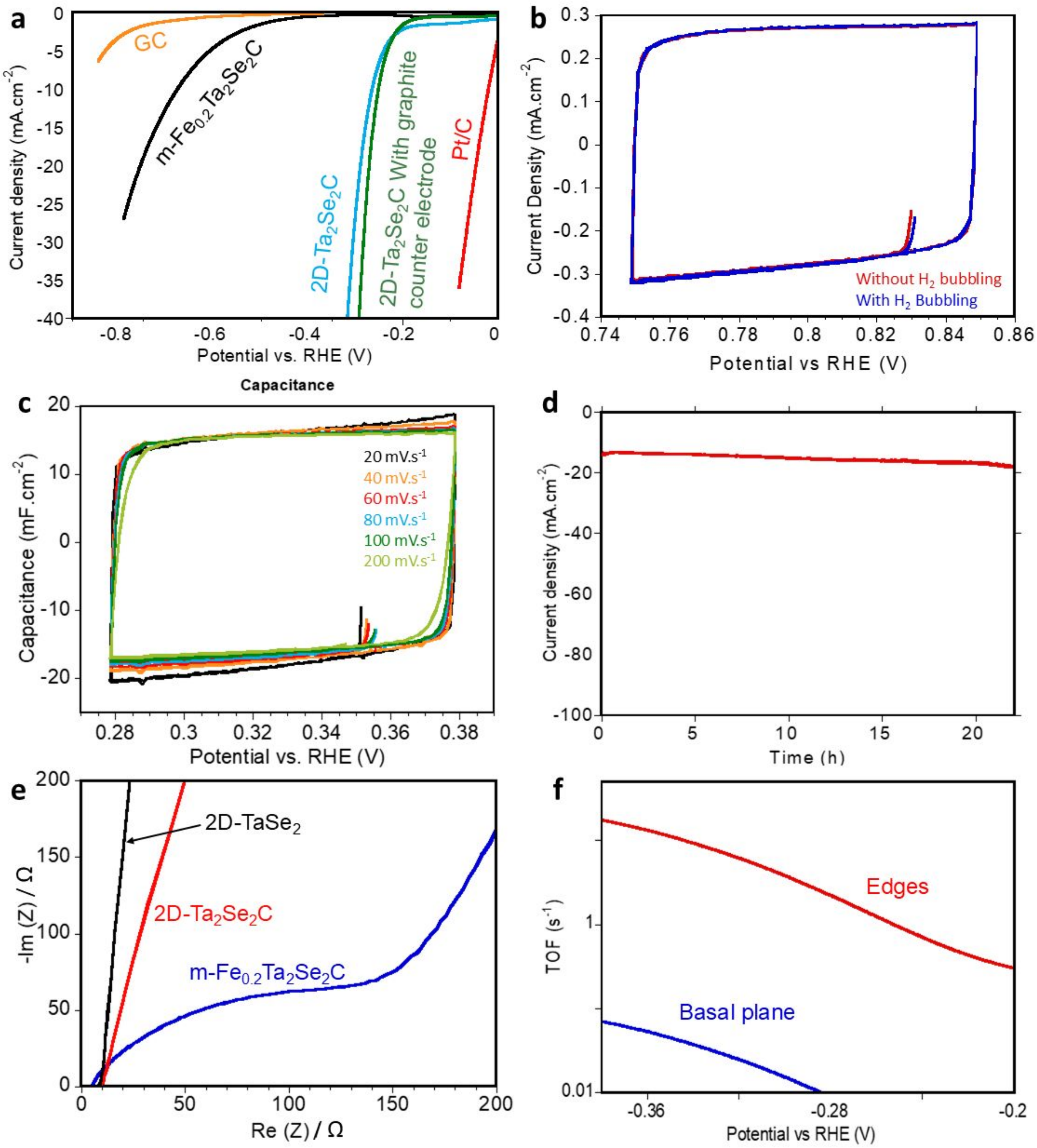

Figure S9: Electrochemical measurements including a) LSV curves for 2D-Ta_2Se_2C with H_2 bubbling after 20 CVs at OCP ± 50 mV in a 3-electrode cell with 0.5 M H_2SO_4 electrolyte with Hg/Hg_2SO_4 in saturated K_2SO_4 as the reference electrode and Pt wire or graphite rod as the counter electrode. b) CVs at 20 mV·s^{-1} scan rate with and without H_2 bubbling for 2D-Ta_2Se_2C, c) capacitance versus the potential for 2D-Ta_2Se_2C calculated using CVs at different scan rates, d) chronoamperometry at a constant potential of -0.3 V, e) PEIS for 2D-Ta_2Se_2C, m-$Fe_{0.2}Ta_2Se_2C$, and 2D-$TaSe_2$, and f) TOF (s^{-1}) versus potential (V) for 2D-Ta_2Se_2C extracted from its LSV graph by considering basal plane (blue) or edges (red) as active sites.

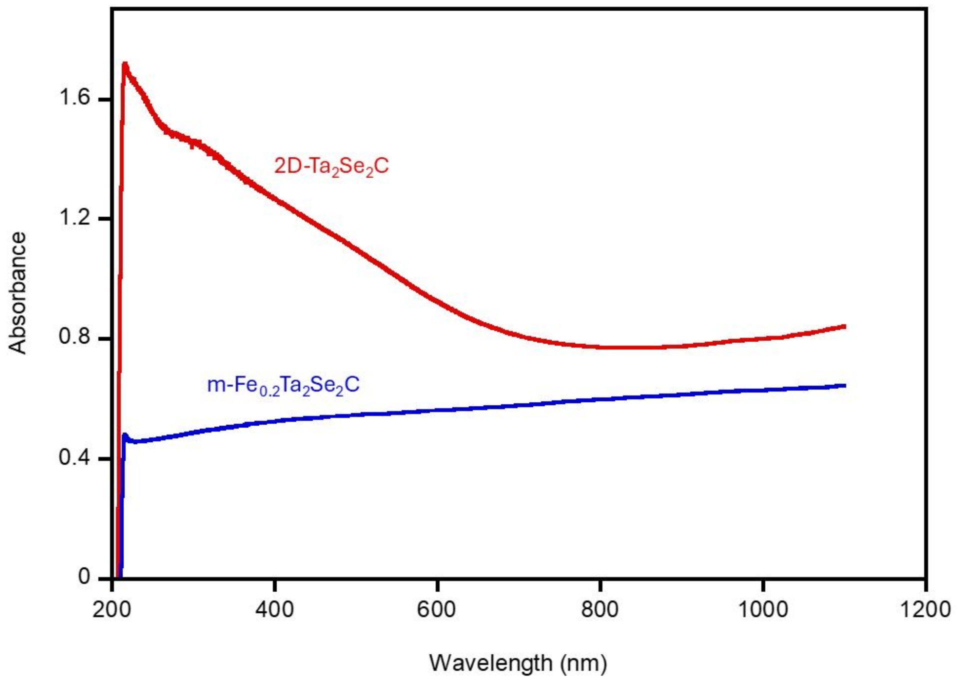

Figure S10: UV-vis spectroscopy graphs of 2D-Ta_2Se_2C and m-$Fe_{0.2}Ta_2Se_2C$ with a 0.6 $g{\cdot}L^{-1}$ concentration.

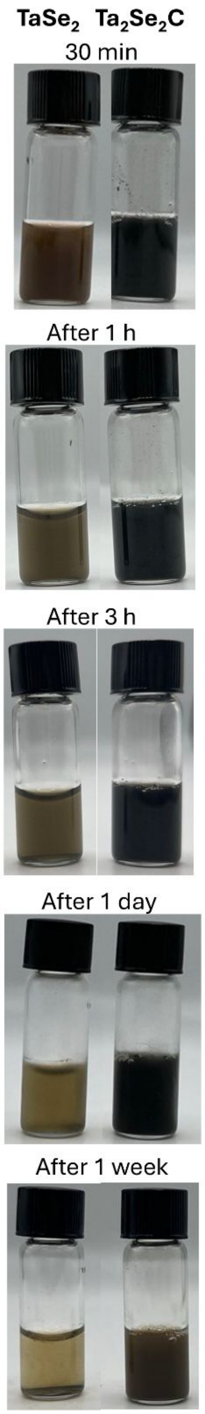

Figure S11: Photographs for 2D-$TaSe_2$ (left) and 2D-Ta_2Se_2C (right) stored in DI water at room temperature.

Theoretical specific surface area calculations for Ta_2Se_2C:

Ignoring the presence of edges and defects, the area of a unit cell that contains one Ta_2Se_2C formula unit = (lattice constant)^2 × sin (60°) = (3.294 Å)^2 × 0.866 = 9.396 × 10^{-20} m^2

The weight of the unit cell = (Ta × 2 + Se × 2 + C) / N_A = (180.948 × 2 + 78.971 × 2 + 12.011) / (6.023 × 10^{23}) g = 8.830 × 10^{-22} g

Specific surface area (one side) = 9.396 × 10^{-20} m^2 / 8.830 × 10^{-22} g = 106.41 m^2/g

Specific surface area (two sides) = 106.41 m^2/g × 2 = 212.82 m^2/g

Calculation of turnover frequency (TOF)[11]

The following equation was used to calculate the average TOF:

TOF = (Number of hydrogen turnovers per geometric surface area) / (Number of sites per geometric area)

Number of hydrogen turnovers per geometric area = [j (mA cm^{-2})] × [1 (C S^{-1}) / 1000 (mA)] × [1 (mol e^{-1}) / 96485 (C)] × [1 (mol H_2) / (2 $mole^{-1}$)] × [6.022 × 10^{23} (H_2 molecules) / 1 (mol H_2)] = 3.12 × 10^{15} × j (mA cm^{-2})

Based on the AFM images, we calculated the average area and perimeter of Ta_2Se_2C flakes. The average area of the flakes was approximately 24,560 nm², and the perimeter was 690 nm.

Using the lattice constant a=3.3068378 Å (or 0.33068378 nm),

the number of unit cells fit within each flake = 24,560 nm² / ($\sqrt{3}/2 \times 0.33068378^2$), resulting in 259,341 unit cells, and the number of unit cells along the perimeter = (690 nm) / 0.33068378 = 2087 unit cells.

Since each unit cell contains 2 selenium (Se) atoms, but only 1 Se atom per unit cell would be exposed on the edge, we determined that each flake has around 2087 Se atoms on its outer edge.

Considering the molar mass of Ta_2Se_2C is approximately 530.83 g.mol^{-1},

the density of Se (atoms.g^{-1}) = (1 g/530.83 g.mol^{-1}) × (6.022×10^{23} atoms/mol) / (259,341 unit cells in flake) × (2087 Se atoms in flake) = 9.13×10^{18} Se atoms.g^{-1}

Finally, to calculate the total number of Se atoms in one gram, we multiplied the total number of unit cells by 2 (since each unit cell contains 2 Se atoms), resulting in approximately 2.26×10^{21} Se atoms. By dividing the number of Se atoms on the edge by the total number of Se atoms, we

obtained a ratio of approximately 0.0040. This means that about 0.4% of the total Se atoms in one gram of Ta_2Se_2C are located on the outer edges of the flakes.

Then we calculated the number of active sites per geometric surface area using the following equation, assuming 0.4% Se sites are active sites:

number of active sites per geometric surface area = m ($mg.cm^{-2}$) × [1 (g) / 1000 (mg)] × [1 (mol Ta_2Se_2C) / 531.83 (g)] × [0.004 × 2 (mol Se) / 1 (mol Ta_2Se_2C)] × [6.022×10^{23} / 1 (mol Se)]

= 9.06×10^{15} × m ($mg.cm^{-2}$)

We used 3.3 μL of a colloidal dispersion of 50 mg mL^{-1}, therefore mass loading (m) was equal to 2.34 mg cm^{-2}. Therefore,

number of active sites per geometric surface area = $9.06 \times 10^{15} \times 2.34$ $mg.cm^{-2}$ = 2.11×10^{16}

If the basal plane was the active surface, then

number of active sites per geometric surface area = $2.11 \times 10^{16} / 0.004 = 5.275 \times 10^{18}$

Table S8. Comparison of the electrocatalytic performance of 2D-Ta_2Se_2C with 2D TMDCs without modification

**Material**	**Overpotential (mV) @ 10 mA.cm^{-2}**	**Tafel Slope (mV.dec^{-1})**	**Turnover Frequency (TOF) (s^{-1})**	**Reference**
2D-MoS_2	330	75	-	[13]
WS_2	~150-200	60-70	0.1 - 0.9	[14]
NbS_2 nanoflake	420	-	-	[15]
CoS_2 nanosheets	398 @ 20 mA.cm^{-2}	148	-	[16]
VS_2 nanoflowers	> 700	133.7	-	[17]
2D-WS_2	380	197	-	[18]
H-TaS_2 film	390	-	-	[19]
2D-TaS_2	200	135	-	[20]
$TaSe_2$ nanobelts	~350-450	70-127	-	[21]
2D-Ta_2Se_2C	264	91	0.1-14	This work

Table S9. ΔG_H* in eV of an H atom on different positions of four TMCC surface and edges

	**Nb_2S_2C**	**Ta_2S_2C**	**Nb_2Se_2C**	**Ta_2Se_2C**
H(1)	0.61	1.22	0.59	0.73
H(2)	0.44	1.04	0.45	0.72
H(3)	0.98	1.34	0.98	1.21
H(4)	0.63	0.43	0.60	0.48
H(5)	-0.32	-0.07	-0.16	0.03

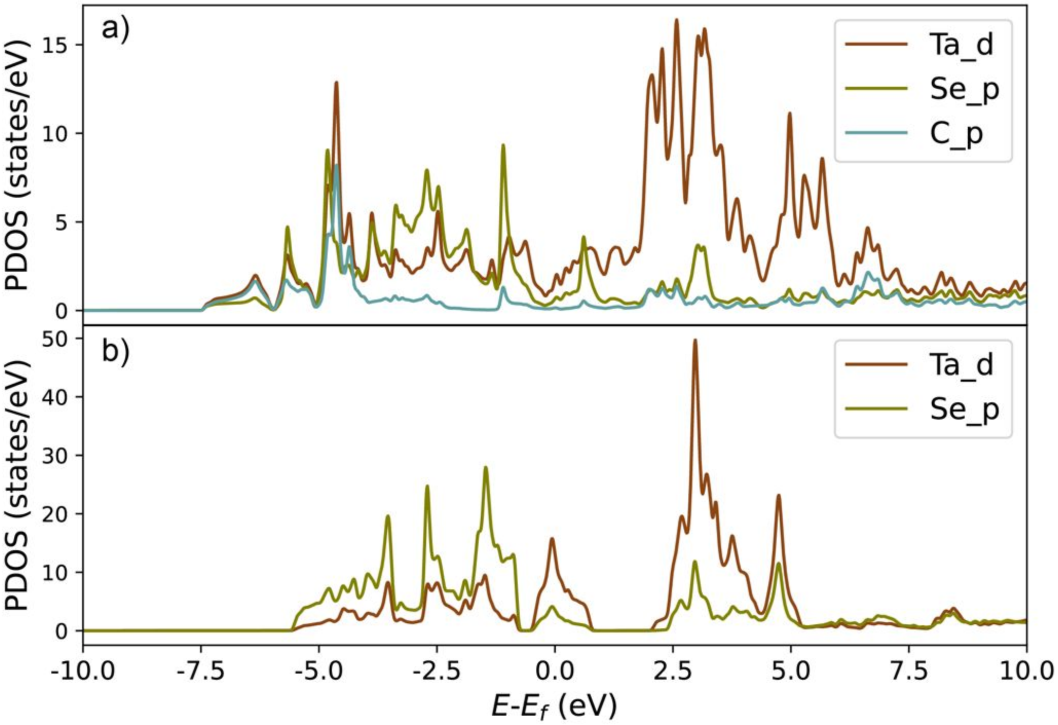

Figure S12. a) PDOS for each element in Ta_2Se_2C. b) PDOS for each element in $TaSe_2$.

References

[1] Yang, W., Cheng, Y., Jiang, M., Jiang, S., Liu, R., Lu, J., Du, L., Li, P. and Wang, C., 2022. Design and fabrication of an ultra-sensitive Ta_2C MXene/Au-coated tilted grating sensor. *Sensors and Actuators B: Chemical*, *369*, p.132391.

[2] Chamlagain, B., Cui, Q., Paudel, S., Cheng, M.M.C., Chen, P.Y. and Zhou, Z., 2017. Thermally oxidized 2D TaS_2 as a high-κ gate dielectric for MoS_2 field-effect transistors. *2D Materials*, *4*(3), p.031002.

[3] Ge, Y., Wang, F., Yang, Y., Xu, Y., Ye, Y., Cai, Y., Zhang, Q., Cai, S., Jiang, D., Liu, X. and Liedberg, B., 2022. Atomically Thin $TaSe_2$ Film as a High-Performance Substrate for Surface-Enhanced Raman Scattering. *Small*, *18*(15), p.2107027.

[4] Deng, Y., Lai, Y., Zhao, X., Wang, X., Zhu, C., Huang, K., Zhu, C., Zhou, J., Zeng, Q., Duan, R. and Fu, Q., 2020. Controlled growth of 3R phase tantalum diselenide and its enhanced superconductivity. *Journal of the American Chemical Society*, *142*(6), pp.2948-2955.

[5] Halim, J., Cook, K.M., Naguib, M., Eklund, P., Gogotsi, Y., Rosen, J. and Barsoum, M.W., 2016. X-ray photoelectron spectroscopy of select multi-layered transition metal carbides (MXenes). *Applied Surface Science*, *362*, pp.406-417.

[6] Martınez, M.T., Callejas, M.A., Benito, A.M., Cochet, M., Seeger, T., Anson, A., Schreiber, J., Gordon, C., Marhic, C., Chauvet, O. and Fierro, J.L.G., 2003. Sensitivity of single wall carbon nanotubes to oxidative processing: structural modification, intercalation and functionalisation. *Carbon*, *41*(12), pp.2247-2256.

[7] Dash, J.K., Chen, L., Dinolfo, P.H., Lu, T.M. and Wang, G.C., 2015. A method toward fabricating semiconducting 3R-NbS_2 ultrathin films. *The Journal of Physical Chemistry C*, *119*(34), pp.19763-19771.

[8] Okpalugo, T.I.T., Papakonstantinou, P., Murphy, H., McLaughlin, J. and Brown, N.M.D., 2005. High-resolution XPS characterization of chemical functionalised MWCNTs and SWCNTs. *Carbon*, *43*(1), pp.153-161.

[9] Xi, Y., Mallavarapu, M. and Naidu, R., 2010. Reduction and adsorption of Pb^{2+} in aqueous solution by nano-zero-valent iron—a SEM, TEM and XPS study. *Materials Research Bulletin*, *45*(10), pp.1361-1367.

[10] Cai, L., 2020. Surface engineering for efficient electrocatalytic water splitting and nitrogen reduction.

[11] Wood, M.A., 2023. The effect of synthesis methods on activity measurements for transition metal phosphides (TMPs) for the hydrogen evolution reaction (HER) (Doctoral dissertation).

[12] Do, H.H., Mahider, T., Phan, N.T., Hong, S.H., Cho, J.H., Ahn, S.H. and Young, K.S., 2021. WS_2–WC–WO_3 nano-hollow spheres as an efficient and durable catalyst for hydrogen evolution reaction. *Nano Convergence*, *8*(1).

[13] Thangasamy, P., Oh, S., Nam, S. and Oh, I.K., 2020. Rose-like MoS_2 nanostructures with a large interlayer spacing of~ 9.9 Å and exfoliated WS_2 nanosheets supported on carbon nanotubes for hydrogen evolution reaction. *Carbon*, 158, pp.216-225.

[14] Voiry, D., Yamaguchi, H., Li, J., Silva, R., Alves, D.C., Fujita, T., Chen, M., Asefa, T., Shenoy, V.B., Eda, G. and Chhowalla, M., 2013. Enhanced catalytic activity in strained chemically exfoliated WS_2 nanosheets for hydrogen evolution. *Nature Materials*, 12(9), pp.850-855.

[15] Najafi, L., Bellani, S., Oropesa-Nuñez, R., Martín-García, B., Prato, M., Mazánek, V., Debellis, D., Lauciello, S., Brescia, R., Sofer, Z. and Bonaccorso, F., 2019. Niobium disulphide (NbS_2)-based (heterogeneous) electrocatalysts for an efficient hydrogen evolution reaction. *Journal of Materials Chemistry A*, *7*(44), pp.25593-25608.

[16] Peng, S., Li, L., Han, X., Sun, W., Srinivasan, M., Mhaisalkar, S.G., Cheng, F., Yan, Q., Chen, J. and Ramakrishna, S., 2014. Cobalt sulfide nanosheet/graphene/carbon nanotube nanocomposites as flexible electrodes for hydrogen evolution. *Angewandte Chemie*, *126*(46), pp.12802-12807.

[17] Chen, X., Yu, K., Shen, Y., Feng, Y. and Zhu, Z., 2017. Synergistic effect of MoS_2 nanosheets and VS_2 for the hydrogen evolution reaction with enhanced humidity-sensing performance. *ACS Applied Materials & interfaces*, *9*(48), pp.42139-42148.

[18] Pan, Y., Zheng, F., Wang, X., Qin, H., Liu, E., Sha, J., Zhao, N., Zhang, P. and Ma, L., 2020. Enhanced electrochemical hydrogen evolution performance of WS_2 nanosheets by Te doping. *Journal of Catalysis*, *382*, pp.204-211.

[19] Najafi, L., Bellani, S., Oropesa-Nuñez, R., Martín-García, B., Prato, M., Pasquale, L., Panda, J.K., Marvan, P., Sofer, Z. and Bonaccorso, F., 2020. TaS_2, $TaSe_2$, and their heterogeneous films as catalysts for the hydrogen evolution reaction. *ACS catalysis*, 10(5), pp.3313-3325.

[20] Li, H., Tan, Y., Liu, P., Guo, C., Luo, M., Han, J., Lin, T., Huang, F. and Chen, M., 2016. Atomic-Sized Pores Enhanced Electrocatalysis of TaS_2 Nanosheets for Hydrogen Evolution. *Advanced Materials (Deerfield Beach, Fla.)*, *28*(40), pp.8945-8949.

[21] Wang, M., Zhang, L., Huang, M., Liu, Y., Zhong, Y., Pan, J., Wang, Y. and Zhu, H., 2020. Morphology-controlled Tantalum Diselenide Structures as Self-optimizing Hydrogen Evolution Catalysts. *Energy & Environmental Materials*, *3*(1), pp.12-18.
